# The effects of psychological interventions on depression and anxiety among Chinese adults with cancer: a meta-analysis of randomized controlled studies

**DOI:** 10.1186/1471-2407-14-956

**Published:** 2014-12-15

**Authors:** Yi-Long Yang, Guo-Yuan Sui, Guang-Cong Liu, De-Sheng Huang, Si-Meng Wang, Lie Wang

**Affiliations:** Department of Social Medicine, China Medical University, 92 North 2nd Road, Heping District, Shenyang 110001 PR China; Department of Environmental Health, School of Public Health, China Medical University, 92 North 2nd Road, Heping District, Shenyang 110001 PR China; Department of Mathematics, School of Basic Medical Science, China Medical University, 92 North 2nd Road, Heping District, Shenyang 110001 PR China; Institute of Hygiene, Zhejiang Academy of Medical Sciences, 182 Tianmushan Road, Hangzhou, 310013 PR China

**Keywords:** Psychological intervention, Ddepression, Anxiety, Chinese adults with cancer, Meta-analysis

## Abstract

**Background:**

Our previous studies found the high prevalence of depression and anxiety among Chinese cancer patients, and many empirical studies have been conducted to evaluate the effects of psychological interventions on depression and anxiety among Chinese cancer patients. This study aimed to conduct a meta-analysis in order to assess the effects of psychological interventions on depression and anxiety in Chinese adults with cancer.

**Methods:**

The four most comprehensive Chinese academic database- CNKI, Wanfang, Vip and CBM databases-were searched from their inception until January 2014. PubMed and Web of Science (SCIE) were also searched from their inception until January 2014 without language restrictions, and an internet search was used. Randomized controlled studies assessing the effects of psychological interventions on depression and anxiety among Chinese adults with cancer were analyzed. Study selection and appraisal were conducted independently by three authors. The pooled random-effects estimates of standardized mean difference (SMD) and 95% confidence intervals (CI) were calculated. Moderator analysis (meta-regression and subgroup analysis) was used to explore reasons for heterogeneity.

**Results:**

We retrieved 147 studies (covering 14,039 patients) that reported 253 experimental-control comparisons. The random effects model showed a significant large effect size for depression (SMD = 1.199, p < 0.001; 95% CI = 1.095-1.303) and anxiety (SMD = 1.298, p < 0.001; 95% CI = 1.187-1.408). Cumulative meta-analysis indicated that sufficient evidence had accumulated since 2000–2001 to confirm the statistically significant effectiveness of psychological interventions on depression and anxiety in Chinese cancer patients. Moderating effects were found for caner type, patients’ selection, intervention format and questionnaires used. In studies that included lung cancer, preselected patients with clear signs of depression/anxiety, adopted individual intervention and used State-Trait Anxiety Inventory (STAI), the effect sizes were larger.

**Conclusions:**

We concluded that psychological interventions in Chinese cancer patients have large effects on depression and anxiety. The findings support that an adequate system should be set up to provide routine psychological interventions for cancer patients in Chinese medical settings. However, because of some clear limitations (heterogeneity and publication bias), these results should be interpreted with caution.

**Electronic supplementary material:**

The online version of this article (doi:10.1186/1471-2407-14-956) contains supplementary material, which is available to authorized users.

## Background

Cancer is considered as a serious and potentially life-threatening illness, and cancer patients have to experience a constellation of challenges, including cancer diagnosis, side effects of medical treatment, sleep disturbance [1], poor adjustment [2], coping strategies [3], emotional distress [4] and problems arising in the family [5]. Therefore, it is well acknowledged that adults diagnosed with cancer are vulnerable to depression and anxiety. In developed countries, such as United States and UK, systemic reviews have indicated that depression and anxiety were two of the common psychological distress in cancer patients [6–9]. Our previous meta-analysis also found that the prevalence of depression (54.90% vs. 17.50%) and anxiety (49.69% vs. 18.37%) were significantly higher in Chinese adults with cancer compared with those without [10]. More seriously, the unrecognized and untreated depression and anxiety could not only lead to difficulty with symptom control, poor compliance with treatment and prolonged recovery time, but also the increased impairment of immune response and impaired quality of life [11–13].

The evidence mentioned above, combined with different national contexts, has led to the increasing interest in psychological interventions in different countries, and cancer patients themselves also reported the need of professional psycho-oncological support [14]. A number of systematic reviews (qualitative and quantitative) have focused on the effectiveness of psychological interventions on depression and anxiety, and psychological interventions, to some extent, have been shown to be effective in reducing depression/anxiety in cancer patients. However, a clear conclusion has not been reached, and the controversy over the effectiveness of psychological interventions still continues. Qualitative review conducted by Newell et al. concluded that no intervention strategy could be recommended for managing depression [15], but Barsevick et al. claimed that psychoeducational interventions were effective for reducing depressive symptoms in cancer patients [16]. Meanwhile, some meta-analyses have provided effect sizes ranging from insignificance [17, 18] to small-medium [19, 20] and small-medium to large [21]. In addition, systematic reviews often focused on either specific type of cancer patients [18] or specific type of intervention [22, 23], which makes it difficult to draw clear conclusions. Recently, Faller et al. pointed out these issues and conducted a comprehensive meta-analysis of 198 controlled studies. The results indicated that psycho-oncologic interventions were effective for depression (Cohen’s d = 0.33, 95% CI = 0.25-0.41) and anxiety (Cohen’s d = 0.38, 95% CI = 0.29-0.46) [20].

Although a number of systematic reviews have been conducted to evaluate the effects of psychological interventions on depression/anxiety in adults with cancer, the effects of psychological interventions on depression/anxiety in Chinese cancer patients have still yet not been examined. Conducting such meta-analysis is vitally important for the following reasons. The first reason is attributed to the number of cancer patients in China. The latest data revealed that China had the world’s largest cancer population (new cases and deaths) in 2012. The numbers of new cases and deaths were 3.07 million (21.8% of world total) and 2.20 million (26.9%) [24]. The second reason is due to the high prevalence of depression and anxiety in Chinese adults with cancer. Compared with the prevalence of depression/anxiety among cancer patients in developed countries, our previous meta-analysis found that the prevalence of depression (54.90%) and anxiety (49.69%) was at a high level in China [10]. Third, although the field of psycho-oncology and its related psychological interventions are relatively young in China, intervention studies and narrative reviews are no longer rare. However, there has not been a comprehensive meta-analysis to assess the effects of psychological interventions on depression/anxiety in Chinese adults with cancer. Forth, because most of the results of these intervention studies were published in Chinese journals, they are usually not easily accessed by other countries’ researchers. Finally, a number of Chinese studies about depression/anxiety of cancer patients adopted psychological interventions (such as cognitive-behavioral and psychoeducational therapy) originated in Western countries. It is necessary to explore whether the psychological interventions widely used in Western countries are also effective among Chinese adults with cancer. More importantly, from a clinical point of view, it would be of practical importance for clinicians to evaluate whether psychological interventions, in addition to the medication, not only have positive effects on depression and anxiety, but also have the possibility of improving the use efficiency of Chinese clinical resources.

The aim of the present meta-analysis, therefore, was to quantify the effectiveness of psychological interventions for treatment of depression and anxiety reported in randomized controlled trials (RCTs) in Chinese adults with cancer. First, we explored the overall effect size of psychological interventions on depression and anxiety in cancer patients. Second, we examined whether the overall effect size was modified by moderating factors (e.g., intervention type, cancer type, and mean age).

## Methods

### Literature search

A systematic search was conducted to identify published literature on the effects of psychological interventions on depression/anxiety in Chinese adults with cancer. The CNKI database (China National Knowledge Infrastructure), Wanfang database, Vip database and CBM database (Chinese Biomedical Literature Database), which are the four most comprehensive Chinese academic databases, were searched from their inception until January 2014. We used ‘depression or depressive disorders or depressive symptoms’ and ‘anxiety or anxiety disorder or anxiety symptoms’ and ‘cancer or oncology or malignant neoplasm or malignant tumor’ combined with ‘psychological intervention or psychological treatment or psychotherapy’ as search themes in the article titles, abstracts and keywords. The reference lists of relevant articles obtained were also screened.

In order to expand searches, PubMed and Web of Science (SCIE) were searched from their inception until January 2014 without language restrictions, and an internet search was also used (e.g., http://www.google.com). The search strategy was: (psychotherapy [MeSH Terms] OR psychotherapy [Title/Abstract] OR psychological therapy [Title/Abstract] OR psychiatric counseling [Title/Abstract] OR psychological intervention [Title/Abstract] OR psychological treatment [Title/Abstract]) AND (neoplasms [MeSH Terms] OR cancer [Title/Abstract] OR neoplasms [Title/Abstract] OR oncology [Title/Abstract]) AND (China [MeSH Terms] OR China or Mainland China [Title/Abstract]) AND (depression [MeSH Terms] OR depressive disorder [MeSH Terms] OR depression [Title/Abstract] OR depressive disorder [Title/Abstract] OR depressive symptoms [Title/Abstract] OR anxiety [MeSH Terms] OR anxiety disorders [MeSH Terms] OR anxiety [Title/Abstract] OR anxiety disorders [Title/Abstract] OR anxiety symptoms [Title/Abstract]).

The screening of the abstracts/titles and full-text articles were performed twice by three authors (YLY, GYS, GCL) independently to reduce reviewer bias and errors.

### Inclusion and exclusion criteria

We included all studies in which: (1) the subjects were aged 16 or older; (2) RCTs were eligible, including experimental group and control group; (3) the subjects were patients diagnosed with cancer; (4) studies were included to those involving more than 30 adults with cancer; (5) a psychological intervention in experimental group was compared to a control group; (6) depression and anxiety were evaluated by well-validated measures, such as clinical diagnosis and self-report questionnaires that previous studies have established the reliability and validity of them as a measure of depression/anxiety at home and abroad; (7) the subjects were from Mainland China (Hong Kong, Taiwan and Macao were excluded due to the long-term influence of foreign culture). We excluded studies in which: (1) the description of psychological interventions was not set forth so clearly in the Method section that other researchers could not duplicate or refer to such studies to conduct psychological interventions; (2) studies in which insufficient data were available to calculate effect sizes were excluded; (3) studies including non-psychological interventions, such as physiotherapy, physical training, and medicine interventions were excluded; (4) Hospice and terminal home care were excluded because they might be distinct from psychological interventions; (5) studies using dimension scores to evaluate depression/anxiety (e.g., depression and anxiety dimension scores of SCL-90) were excluded. Eligibility judgment and data extraction were recorded and carried out independently by two authors (YLY and GYS) in a standardized manner. Any disagreements with them were resolved by discussion and the involvement of another author (LW).

### Quality assessment

Although many scales are used to evaluate the methodological quality of RCTs, none can provide an adequately and comprehensively reliable assessment [25]. A systematic review indicated that Jadad scale presented the best validity and reliability evidence compared with other scales [25], but Jadad scale only including 3 items [26] may be too simple to well assess quality of RCTs in our meta-analysis. Therefore, the modified Jadad scale for assessing quality of RCTs was adapted for use [27]. The modified Jadad scale is an eight-item scale designed to assess randomization, blinding, withdrawals/dropouts, inclusion/exclusion criteria, adverse effects, and statistical analysis. In this meta-analysis, blinding (2 points) and adverse effects (1 point) were excluded, because blinding is often not feasible for trials of psychological interventions, and psychological interventions usually has few negative side effects. As a result, the score for each study can range from 0 (lowest quality) to 5 (highest quality).We defined three categories: the study was considered to have high quality (low risk of bias) if it scored 4 points or above, studies that scored 1 point or below were categorized as having low quality (high risk of bias), studies that scored 2 points or 3 points were considered as having medium quality (moderate risk of bias). Any disagreements with authors (GCL and SMW) were resolved by discussion and the involvement of another author (LW).

### Data extraction

A standardized data extraction scheme was developed and pilot tested on 5 included studies. For all studies, two authors independently extracted data (DSH and SMW). Disagreements were resolved by discussion. In situations where the coder was unsure, one of the authors was consulted until consensus was reached.

Data extracted from the present study included author name, year of publication, age range and mean age, simple size, outcomes (depression and anxiety) and assessment instruments (clinical diagnosis/self-report), selection of participants by the clear signs of depression/anxiety, cancer type, cancer stage, intervention type (cognitive-behavioral interventions (CBT), patients education (PE), relaxation/imagery, social/family support, music therapy, nursing intervention, other), professionalism of therapists (e.g., nurse, doctor, and psychologist), intervention format (individual, group, family), information about treatments and timing of assessment, and mean and standard deviation (SD) of each study.

Among these types of interventions, the seven categories were defined as follows. CBT included cognitive, cognitive-behavioral, and behavioral methods focused on changing specific thoughts or behaviors or on learning specific coping skills. PE (or called information and counseling) included interventions primarily providing health education (procedural or medical information), coping skills training, stress management, and psychological support. If interventions mainly focused on coping skills or psychological support, these were classified as “CBT” or “social/family support”. Relaxation and imagery techniques were any method, process, or activity that helped patients to relax and attain a state of calmness. Social/family support referred to nonprofessionally/professionally guided support groups (social support) or to the patients’ family members (family support) that provided mutual help and support (e.g., emotional support, financial support, and the communication of shared experiences). Music therapy referred to an interpersonal process in which the therapist used music and all of its facets (physical, emotional, social, and aesthetic) to help patients to improve or maintain their health, and it should be different from “relaxation/imagery” when conducted as the only intervention. Nursing intervention were the actions undertaken by caregivers (mainly nurse) to adopt nonspecific interventions to further provide a high level of care, such as promoting communication with patients and their families, understanding, encouraging and comforting patients, strengthening nursing care, and providing suitable environment. If interventions aimed at emotional support and emotional release, these were classified as “social/family support” or “relaxation/imagery”. Interventions not matching these definitions were classified as “other”.

### Meta-analysis

#### Assessment of overall effect size

We computed the effect size of standardized mean difference (SMD) for each study by subtracting the average post-test score of the control group from that of the experimental group and dividing the result by the pooled standard deviations of the experimental group and control group. Means and standard deviations of depression/anxiety were used for computation of SMD (Cohen’s *d*). A SMD of 1 indicates a relatively stronger improvement in experimental group by one standard deviation larger than the mean of the control group. For a certain outcome, only one effect size per study was included. If an experimental-control comparison provided more than one effect size for depression/anxiety, the results were averaged. The pooled random-effects estimates of SMD and 95% confidence intervals (CI) were used as the summary measure of effect. A random effects model was used because it involves the assumption of statistical heterogeneity between studies [28]. Effect sizes of 0.80 are regarded as large, while effect sizes of 0.50 are moderate, and effect sizes of 0.2 are small [29]. A two-tailed P value of less than 0.05 was considered to be significant. Overall effects were analyzed using the statistical software Stata v11.0.

#### Assessment of heterogeneity

Heterogeneity was evaluated with the Q statistic and I^2^ statistic. The Q statistic is used to assess whether differences in results are compatible with chance alone. If the P value of Q statistic is above 0.05, it indicates that there is no significant heterogeneity, but the Q statistic is sensitive to the number of studies [30]. To complement the Q statistics, the I^2^ statistic which denotes the variance among studies as a proportion of the total variance was also calculated and reported, because I^2^ is not sensitive to the number of studies [30]. Larger values of I^2^ show increasing heterogeneity. An I^2^ of 0% shows no observed heterogeneity, while 25% shows low, 50% moderate, and 75% high levels of heterogeneity [31].

#### Moderator analyses

When the hypothesis of homogeneity was rejected by the Q statistic and I^2^ statistic, meta-regression (continuous variable) and subgroup analysis (categorical variable) were conducted in order to explore the potential moderating factors for heterogeneity [30]. In our study, meta-regression and subgroup analysis were conducted for moderating factors, including cancer type, cancer stage (early vs. advanced stage), patients’ selection (clear signs of depression/anxiety vs. regardless of depression/anxiety level), patients’ age, simple size, quality of study, intervention type (CBT, PE, relaxation/imagery, social/family support, music therapy, nursing intervention, other), intervention format (individual vs. other formats), appropriate randomization (yes/no), the used questionnaires and timing of assessment. Because most of studies in our meta-analysis included more than one type of intervention, intervention type was not considered as a categorical variable, and the sum types of intervention was the indicator of intervention type.

#### Assessment of publication bias

The potential of publication bias of the included studies was first examined by funnel plot symmetry. A funnel plot is a useful graph designed to check the existence of publication bias in meta-analyses. A symmetric funnel shape indicates that publication bias is unlikely, but an asymmetric funnel suggests the possibility of publication bias. However, some authors have argued that visual interpretation of funnel plots is too subjective to be useful [32]. So Begg’s test and Egger’s test were further used to more objectively test for its presence (as implemented in Stata v11) [33, 34].

### Cumulative meta-analysis

We explored the evolution of evidence of the effects of psychological interventions on depression and anxiety among Chinese cancer patients over time using cumulative meta-analysis [35]. Studies were sequentially accumulated by year they first became available (e.g., publication in a journal) to a random-effects model using the “metacum” user-written command in Stata v 11.

## Results

### Study selection

**Figure 1:**
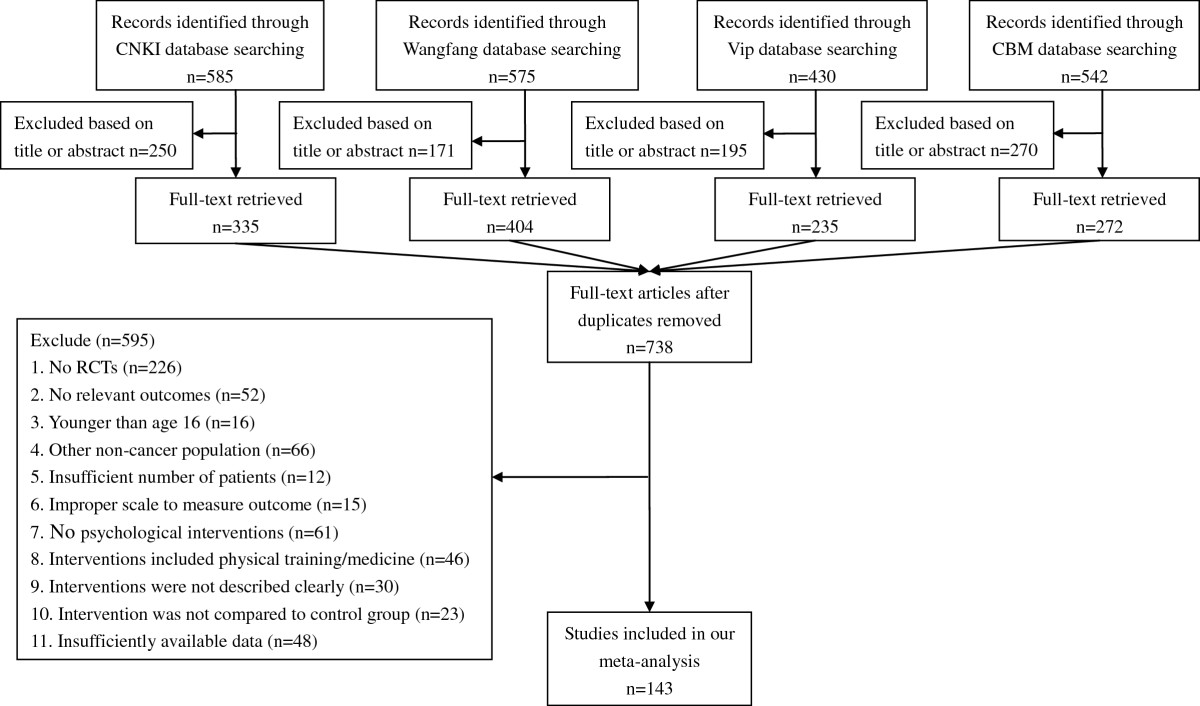
**Selection process of studies for the meta-analysis (Chinese databases).** Abbreviations: RCTs, randomized controlled trials; CNKI, China National Knowledge Infrastructure; CBM, Chinese Biomedical Literature Database.

A flowchart describing the inclusion and exclusion process was presented. As shown in Figure 1, we identified the possibly eligible articles through CNKI database (n = 585), Wangfang database (n = 575), Vip database (n = 430) and CBM database (n = 542). The titles and abstracts of these articles were respectively studied by the three authors (YLY, GYS and GCL), and the full-text articles without duplicates (n = 738) were selected for further examination. Based on the full-text of these 738 studies, 595 did not meet the inclusion criteria as documented in Figure 1. In total, 143 studies reporting on 247 experimental-control comparisons (Depression: n = 119; Anxiety: n = 128) were included in the present meta-analysis [36–178].

**Figure 2:**
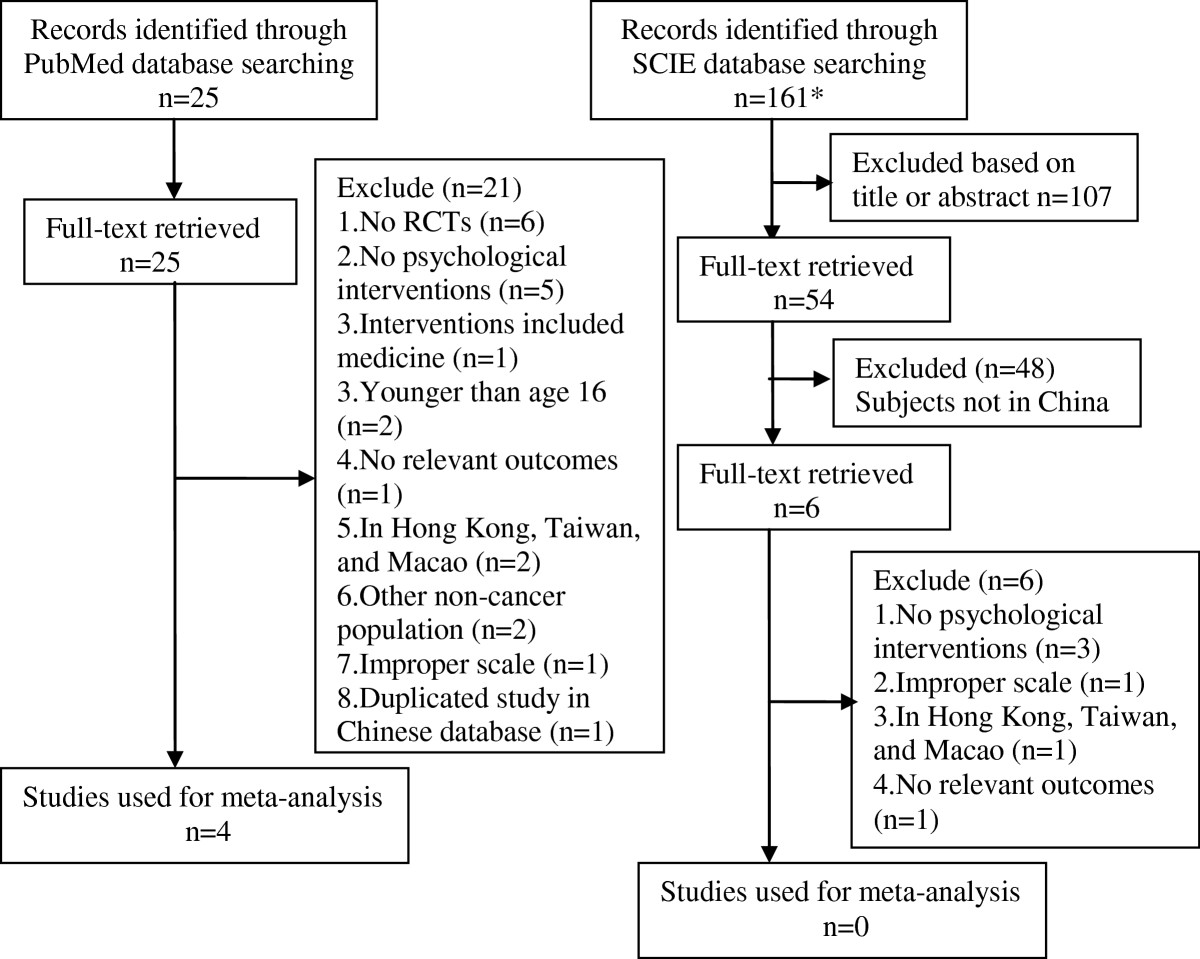
**Selection process of studies for the meta-analysis (international databases).** Abbreviations: RCTs, randomized controlled trials; SCIE, Web of Science. *Records could not be identified through the original search strategy, and “China [MeSH] OR China [Title/Abstract] or Mainland China [Title/Abstract]” was excluded from the original search strategy.

In order to expand searches, we also searched the international databases of PubMed, SCIE (as shown in Figure 2), and an internet search (e.g., http://www.google.com). There were 4 studies from PubMed that met our inclusion criteria through the international databases search [179–182].

### Characteristics of included studies

Study characteristics were listed in Table 1. The studies of this meta-analysis, including 133 journal articles and 14 dissertations, were published from 2000 to 2013. The studies comprised 14,039 subjects. The mean sample size was 95.5 (median: 80; range: 30–326). Subjects had a mean age of 52.4 years (median: 51.9; range: 39–74). Depression and anxiety were assessed by clinical diagnosis in 16 studies [37, 42, 47, 48, 58, 83, 101, 102, 107, 108, 113, 127, 132, 144, 146, 181], while that of the other studies was assessed by self-report questionnaires like Self-rating Depression Scale (SDS) and Self-rating Anxiety Scale (SAS). For a certain outcome, each study only included one effect size. Only 15% of studies preselected patients according to their clear signs of depression/anxiety. Forty-six percent included mixed cancer diagnoses, and 15% included breast cancer and gynaecological cancer, respectively. Seventeen percent of studies included advanced cancer patients, and 6% included early cancer patients. PE (74%) was the most common intervention type used, and the proportion on the order was social/family support (63%), CBT (54%), relaxation/imagery (54%), nursing intervention (52%), music therapy (14%), and other interventions (14%). Therapists included nurses (46%), doctor and oncologist (14%), psychologists (11%), and others. Finally, 21% of studies only employed the individual (i.e., one-on-one) intervention format and 68% clearly provided the information about treatments.

**Table 1:** **Characteristics of the included studies**

Author & years	Age (Mean)	Subjects (n1 + n2)	Outcomes	Patients’ selection	Cancer type	Cancer stage	Intervention type	Therapist	Intervention format
Wang et al. 2000 [39]	18-67	38 + 38	Both (SDS,SAS)	Nonselective	Mixed	-	③	Doctor	A
Zhao et al. 2000 [153]	22-67 (52)	42 + 41	Both (SDS,SAS)	Nonselective	Mixed	Advanced	① + ② + ③ + ④	-	A + B + C
Cai et al. 2001 [144]	26-70 (50.6)	116+ 46	Both (HAMD,SAS)	Nonselective	Mixed	-	⑤	-	A + B
Yang et al. 2002 [42]	28-65 (44.6)	34 + 30	Depression (HAMD)	Nonselective	Mixed	-	① + ③	-	B
Guan et al. 2002 [123]	30-71	44 + 44	Both (SDS,SAS)	Nonselective	Mixed	-	② + ③ + ④ + ⑦	Oncologist	B
Li et al. 2002 [76]	32-71 (51.2)	61 + 47	Both (SDS,STAI)	Nonselective	Mixed	Early	① + ② + ③ + ④ + ⑥	-	A + B + C
Lian et al. 2003 [44]	18-65 (46)	50 + 50	Both (SDS,SAS)	Nonselective	Head/neck	-	① + ② + ④	-	-
Wu & Wang 2003 [148]	30-78 (56)	63 + 57	Both (SDS,SAS)	Nonselective	Lung	Advanced	① + ② + ③ + ④	Doctor (training)	A + B + C
Zhong et al. 2003 [38]	>16	91 + 92	Both (SDS,SAS)	Nonselective	Mixed	-	②	-	-
Lou et al. 2003 [101]	31-72	85 + 86	Depression (DSI)	Nonselective	Mixed	-	① + ② + ④	Nurse	A + C
Xu 2004 [115]	30-70 (58)	150 + 100	Both (SDS,SAS)	Nonselective	Digestive tract	-	② + ④ + ⑥ + ⑦	Nurse	A + C
Wang 2004 [93]	36-65	30 + 22	Depression (SDS)	Nonselective	Breast	-	② + ③ + ④ + ⑥	Nurse	A + C
Bu et al. 2005 [155]	>18 (46.5)	30 + 30	Anxiety (SAS)	Selective	Digestive tract	-	② + ③ + ④ + ⑥	Nurse	-
Lou et al. 2005 [164]	24-71 (58)	75 + 75	Anxiety (SAS)	Nonselective	Mixed	-	① + ② + ③ + ④	-	A + C
Liu et al. 2006 [143]	16-77 (51.9)	58 + 53	Both (SDS,STAI)	Nonselective	Mixed	-	⑤	-	A
Cheng et al. 2006 [107]	>16 (65.3)	15 + 15	Both (HAMD,HAMA)	Nonselective	Mixed	Advanced	① + ③ + ⑥ + ⑦	-	-
Wang et al. 2006 [75]	>18 (56.1)	31 + 31	Both (SDS,SAS)	Nonselective	Mixed	Advanced	① + ④	Nurse (training)/Oncologist	B
Ni et al. 2007 [165]	>18 (55.4)	169 + 157	Anxiety (SAS)	Nonselective	Mixed	Advanced	① + ② + ④ + ⑦	Doctor	A + C
Pang & Wang 2007 [166]	31-62 (59)	43 + 42	Anxiety (SAS)	Nonselective	Breast	-	② + ③ + ④ + ⑥	Nurse (training)	A + C*
Qian & Cai 2007 [50]	18-65	40 + 40	Both (SDS,SAS)	Nonselective	Gynecology	-	① + ② + ③ + ④ + ⑥	Nurse	A + B + C
Wen & Liang 2007 [69]	16-40	73 + 63	Both (SDS,STAI)	Nonselective	Mixed	-	① + ③ + ④ + ⑥	-	A + B
Kang 2007 [128]	40-60	30 + 30	Both (SDS,SAS)	Nonselective	Breast	Advanced	② + ⑤	-	A + B
Zheng et al. 2007 [109]	39-86 (58)	35 + 35	Both (SDS,SAS)	Selective	Mixed	-	① + ② + ④ + ⑤	Oncologist/Nurse	A + C
Deng et al. 2007 [110]	32-70 (55.3)	60 + 60	Both (SDS,SAS)	Nonselective	Mixed	-	② + ③ + ④	Doctor	A + C
Xing 2007 [103]	43-75 (57.2)	50 + 50	Both (SDS,SAS)	Nonselective	Gynecology	-	② + ③ + ④ + ⑥	-	A + C
Wu et al. 2007 [59]	18-70 (48.4)	40 + 40	Both (SDS,SAS)	Nonselective	Mixed	Advanced	① + ② + ③ + ④ + ⑥	Nurse	A + B + C
Xu 2007 [88]	20-70	32 + 32	Both (SDS,SAS)	Nonselective	Gynecology	-	① + ③ + ④ + ⑥	Nurse	A + B + C
Han & Liu 2007 [151]	27-76 (59.1)	30 + 30	Both (SDS,SAS)	Nonselective	Mixed	-	① + ② + ④ + ⑤	Nurse	A + B
Huang et al. 2008 [54]	>16	40 + 40	Both (SDS,SAS)	Nonselective	Mixed	-	① + ② + ③ + ④ + ⑤	Nurse	A
Zheng et al. 2008 [116]	>18 (58.9)	38 + 39	Both (SDS,SAS)	Nonselective	Mixed	-	① + ② + ③ + ④	-	A + C
Yang 2008 [79]	>16	40 + 40	Both (SDS,SAS)	Nonselective	Gynecology	-	① + ② + ③ + ④	Nurse	A + C
Han 2008 [160]	33-65 (48.1)	32 + 35	Anxiety (SAS)	Nonselective	Breast	-	① + ② + ④ + ⑥	Nurse	A + C
Jiang et al. 2008 [161]	28-64 (52)	52 + 52	Anxiety (SAS)	Nonselective	Mixed	-	③	-	A
Li et al. 2008 [163]	25-78 (53)	24 + 24	Anxiety (SAS)	Nonselective	Digestive tract	-	⑥	Nurse	A
Wang et al. 2008 [169]	>18	40 + 40	Anxiety (SAS)	Nonselective	Digestive tract	Early	① + ② + ③ + ④ + ⑥	-	-
Ji 2008 [106]	22-83 (54.2)	40 + 40	Depression (SDS)	Nonselective	Mixed	-	① + ② + ③ + ④	Doctor/Nurse (training)	A + B + C
Jin & Zhu 2008 [122]	42-65 (59)	30 + 30	Both (SDS,SAS)	Nonselective	Lung	-	① + ② + ③ + ④ + ⑥	-	A + B
Li et al. 2008 [99]	26-73 (43.7)	30 + 30	Both (SDS,SAS)	Nonselective	Digestive tract	-	② + ④ + ⑥ + ⑦	Nurse	A + C
Liu et al. 2008 [52]	24-70 (50)	90 + 50	Both (SDS,SAS)	Nonselective	Gynecology	-	① + ② + ④	-	A + B + C
Yang 2008 [136]	18-70 (49.7)	31 + 31	Both (SDS,SAS)	Nonselective	Breast	Early	① + ④ + ⑥ + ⑦	Clinical psychologist	B
Zhou 2008 [100]	26-57	32 + 32	Both (SDS,SAS)	Nonselective	Blood	-	② + ③ + ④ + ⑥	Nurse/Psychologist	-
Mao et al. 2008 [113]	>16 (55.3)	82 + 76	Both (HAMD,HAMA)	Nonselective	Mixed	-	② + ⑥	Nurse	-
Liu 2008 [132]	25-72	31 + 31	Both (HAMD,HAMA)	Nonselective	Gynecology	Advanced	② + ③	-	A
Zheng et al. 2008 [125]	18-70 (51.4)	50 + 50	Both (SDS,SAS)	Nonselective	Mixed	Advanced	① + ② + ⑦	Nurse	A
Chen et al. 2009 [156]	>18	33 + 32	Anxiety (SAS)	Selective	Digestive tract	-	① + ② + ③ + ④ + ⑥ + ⑦	Psychologist/Nurse (training)	A
Li 2009 [89]	30-60 (46)	30 + 30	Both (SDS,SAS)	Nonselective	Gynecology	-	① + ③ + ④ + ⑤	-	-
Li et al. 2009 [78]	22-84	78 + 78	Both (SDS,SAS)	Nonselective	Digestive tract	-	① + ② + ④	Psychologist/Doctor/Nurse	A + B + C
Fu et al. 2009 [145]	26-60 (39.3)	40 + 38	Both (SDS,SAS)	Nonselective	Breast	Advanced	⑤	-	A
Qiu 2009 [133]	30-70	30 + 30	Both (SDS,SAS)	Nonselective	Lung	-	① + ② + ③	-	A
Sun 2009 [134]	>18 (43.4)	30 + 30	Both (HASD)	Nonselective	Mixed	-	① + ②	-	A
Xia 2009 [118]	24-60 (47)	28 + 28	Both (SDS,SAS)	Selective	Mixed	-	① + ② + ③ + ⑥	Nurse (training)	-
Zhang 2009 [139]	18-70 (55)	34 + 32	Depression (SDS)	Selective	Mixed	-	① + ② + ③	-	A
Zhou 2009 [140]	18-55 (45.9)	30 + 30	Both (SDS,SAS)	Selective	Breast	Early	⑤	-	A
Li et al. 2009 [63]	18-72 (40.5)	61 + 59	Both (SDS,SAS)	Nonselective	Head/neck	-	① + ② + ③ + ④	Psychologist	B
Geng et al. 2010 [104]	23-82	124 + 123	Both (SDS,SAS)	Nonselective	Mixed	-	① + ② + ③ + ④+ ⑥	Researcher (training)	A + C
Zhan & Cheng 2010 [105]	18-75	35 + 35	Both (SDS,SAS)	Nonselective	Lung	Advanced	① + ② + ③ + ④	Doctor/Nurse (training)	A + B + C
Cheng et al. 2010 [45]	21-69 (47)	50 + 50	Both (SDS,SAS)	Nonselective	Head/neck	-	② + ③ + ④ + ⑤	Oncologist/Psychologist/Nurse	A + B + C
Li et al. 2010 [91]	41-68 (52.2)	50 + 50	Both (SDS,SAS)	Nonselective	Gynecology	-	② + ③ + ⑥	Nurse	-
Guan et al. 2010 [81]	38-70 (44)	30 + 30	Both (SDS,SAS)	Nonselective	Urinary	-	② + ③ + ④	Nurse	-
Li 2010 [111]	31-72 (49.7)	57 + 57	Both (SDS,SAS)	Nonselective	Mixed	Advanced	④ + ⑥	Doctor/Nurse	A + B + C
Zhang 2010 [138]	>18 (49.7)	47 + 48	Both (SDS,SAS)	Nonselective	Breast	Early	①+④+⑦	-	B
Su & Wang 2010 [167]	>18 (52.9)	41 + 46	Anxiety (SAS)	Selective	Digestive tract	-	①+②+④+⑥	Nurse (training)	A + C
Fu et al. 2010 [159]	27-64 (46.5)	36 + 28	Anxiety (SAS)	Nonselective	Mixed	Advanced	②+ ③+ ④+ ⑤+ ⑥	-	A + C
Wu & Zhang 2010 [170]	30-75 (48)	40 + 39	Anxiety (SAI)	Nonselective	Digestive tract	Advanced	① + ③ + ④ + ⑤ + ⑥	Nurse	-
Zhou 2010 [176]	30-65	60 + 60	Anxiety (SAS)	Nonselective	Breast	-	① + ② + ③ + ④ + ⑥	Nurse	-
You et al. 2010 [171]	>18 (48.9)	33 + 29	Anxiety (SAS)	Nonselective	Breast	-	① + ②	Nurse	A
Ren et al. 2010 [154]	33-74 (54.2)	40 + 37	Both (SDS,SAS)	Nonselective	Mixed	-	① + ③ + ④	-	A + B + C
Xu 2010 [40]	27-73 (51)	47 + 43	Both (SDS,SAS)	Selective	Mixed	-	① + ② + ⑥	Nurse	A
Guo et al. 2010 [71]	23-82 (45.4)	45 + 45	Both (SDS,SAS)	Nonselective	Mixed	Advanced	① + ②	Researcher (cognitive therapy training)	A
Tang et al. 2010 [126]	>18 (49.8)	40 + 40	Both (SDS,SAS)	Nonselective	Breast	-	① + ② + ③ + ④	Nurse	A + C
Liu et al. 2010 [46]	>16 (51.1)	50 + 50	Both (SDS,SAS)	Nonselective	Mixed	-	② + ⑥	Nurse (training)	-
Shi et al. 2010 [108]	21-79 (54)	20 + 20	Depression (HAMD)	Selective	Digestive tract	Advanced	④ + ⑥	Psychologist	A + B
Liu et al. 2010 [87]	>16 (57.5)	37 + 35	Both (SDS,SAS)	Nonselective	Lung	Early	②+④	Psychologist	A + B + C
Wang 2010 [90]	>16 (48.1)	43 + 43	Both (SDS,SAS)	Nonselective	Gynecology	-	① + ② + ③ + ④ + ⑥	-	A + B
Huang et al. 2010 [86]	>16 (63.6)	32 + 28	Both (SDS,SAS)	Nonselective	Lung	-	① + ② + ④ + ⑥	-	-
Zhang & Yu 2011 [94]	21-53	60 + 60	Both (SDS,SAS)	Nonselective	Breast	-	② + ③ + ④ + ⑥	-	A + C
Du et al. 2011 [61]	>16 (42.7)	28 + 30	Both (SDS,SAS)	Nonselective	Breast	-	①	Nurse	A
Li et al. 2011 [149]	>18 (47)	20 + 20	Both (SDS,SAS)	Selective	Gynecology	-	① + ② + ③ + ④ + ⑥	-	A
Zhou et al. 2011 [180]	25-65 (45)	54 + 51	Depression (SDS)	Nonselective	Breast	-	⑤	-	A
Liu 2011 [131]	23-65	30 + 30	Both (SDS,SAS)	Selective	Gynecology	-	①+③+⑤+⑦	-	A + C
Shen et al. 2011 [64]	39-71 (58.1)	37 + 38	Both (SDS,SAS)	Nonselective	Digestive tract	-	②+⑥	Nurse	A
Zhu et al. 2011 [65]	>60 (74)	50 + 48	Depression (SDS)	Nonselective	Digestive tract	-	② + ③ + ④ + ⑥	Nurse	A + B
Meng et al. 2011 [95]	34-74 (57)	46 + 41	Both (SDS,SAS)	Nonselective	Mixed	Advanced	② + ③ + ⑥ + ⑦	Nurse	-
Dai et al. 2011 [157]	23-78 (57.9)	66 + 68	Anxiety (SAI)	Nonselective	Mixed	-	① + ② + ③ + ④ + ⑥	Oncologist/Psychologist/Nurse/Nutritionist	B
Jiao et al. 2011 [162]	40-66 (55.8)	34 + 34	Anxiety (SAS)	Nonselective	Gynecology	Advanced	① + ② + ③ + ④ + ⑥	Nurse (training)	A + C
Ye 2011 [56]	24-65 (43.5)	20 + 20	Both (SDS,SAS)	Selective	Gynecology	-	①+③	-	A
Li 2011 [130]	18-80	37 + 32	Depression (SDS)	Nonselective	Digestive tract	Early	① + ② + ③ + ④ + ⑤ + ⑥ + ⑦	-	A + B + C
Liu et al. 2011 [41]	30-50	50 + 50	Depression (SDS)	Nonselective	Mixed	-	① + ② + ④	Medical staff	A + B
Wang et al. 2011 [37]	>16 (59.03)	30 + 31	Both (HAMD,HAMA)	Nonselective	Mixed	-	⑤	Psychologist	B
Cao2011 [173]	>18	30 + 30	Anxiety (SAS)	Nonselective	Breast	-	③	Nurse (psychological/music training)	A
Zhao & Zhang 2011 [174]	18-70	21 + 20	Anxiety (SAS)	Selective	Gynecology	-	① + ② + ③ + ④ + ⑥	Nurse	A + C
Cao & Li 2011 [67]	>16	55 + 53	Both (SDS,SAS)	Nonselective	Mixed	-	③	-	A + B + C
Huang et al. 2011 [152]	33-71	140 + 139	Depression (SDS)	Nonselective	Mixed	-	① + ② + ⑥	Nurse (training)	A + B + C
Hu & Yan 2011 [62]	30-52 (45)	32 + 32	Both (SDS,SAS)	Nonselective	Mixed	-	① + ④	Psychologist/Oncologist/Nurse (training)	B
Guan & Jin 2011 [120]	18-75 (66)	78 + 78	Both (SDS,SAS)	Nonselective	Mixed	Advanced	① + ② + ③ + ④ + ⑥ + ⑦	-	A + C
Lv et al. 2011 [77]	25-65	38 + 38	Both (SDS,SAS)	Nonselective	Gynecology	Early	① + ② + ③ + ④	Nurse	A + B
Li et al. 2011 [182]	25-65 (45)	54 + 51	Anxiety (SAI)	Nonselective	Breast	-	⑤	-	A
Cao & Jiang 2011 [177]	>18 (51.5)	42 + 42	Anxiety (SAS)	Nonselective	Lung	-	② + ③ + ④ + ⑥	Nurse	A + B + C
Huang2011 [102]	>16 (54.23)	40 + 40	Depression (HAMD)	Selective	Mixed	-	① + ② + ③ + ⑥	-	A + B
Zheng et al. 2011 [141]	21-81 (54)	102 + 111	Both (SDS,SAS)	Nonselective	Mixed	-	②+④+⑥	Nurse	A + B + C
Wu & Dong 2011 [47]	48-78 (63.3)	33 + 33	Both (HAMD,HAMA)	Selective	Mixed	-	② + ③ + ④ + ⑤	-	A + C
Zheng et al. 2012 [150]	19-70 (52.6)	28 + 28	Depression (SDS)	Selective	Mixed	-	① + ② + ④	Doctor	A + C
Wang & Xiao 2012 [124]	>18 (57.5)	42 + 42	Both (SDS,SAS)	Nonselective	Mixed	-	① + ② + ⑥	Psychologist	A
Wei 2012 [135]	>18 (48.1)	30 + 30	Both (SDS,SAS)	Nonselective	Breast	-	① + ② + ③ + ⑥	-	A + B
Feng 2012 [55]	35-65 (50.9)	45 + 45	Both (SDS,SAS)	Nonselective	Breast	-	① + ② + ⑦	-	A
Yang et al. 2012 [73]	48-81	20 + 20	Both (SDS,SAS)	Nonselective	Breast	Advanced	① + ② + ③ + ④	Psychologist	A + B + C
Zhao et al. 2012 [84]	18-75 (57.2)	103 + 102	Both (SDS,SAS)	Nonselective	Mixed	-	① + ② + ⑦	Doctor	-
Gu 2012 [121]	47-74 (64.6)	52 + 48	Both (SDS,SAS)	Nonselective	Lung	-	④ + ⑥ + ⑦	Nurse	A + C
Zheng2012 [58]	45-72 (51.6)	30 + 30	Both (HAMD,HAMA)	Selective	Mixed	-	② + ③ + ⑥	-	-
Yang2012 [66]	59-76 (65.4)	23 + 20	Depression (SDS)	Selective	Digestive tract	-	②+④+⑥	Nurse	A + C
Sun et al. 2012 [60]	21-78 (49.4)	89 + 89	Both (SDS,SAS)	Nonselective	Mixed	-	② + ③ + ④ + ⑥	Psychologist/Nurse	A + B + C
Liu et al. 2012 [98]	>18 (48.6)	30 + 30	Both (SDS,SAS)	Nonselective	Digestive tract	-	③ + ④+ ⑦	-	A + C
Yang et al. 2012 [70]	20-70 (58.4)	48 + 40	Both (SDS,SAS)	Nonselective	Mixed	Advanced	② + ④ + ⑥	Nurse	-
Li 2012 [129]	34-36 (41.7)	51 + 51	Both (CES-D,SAI)	Nonselective	Mixed	-	② + ⑥	Nurse	A
Zhu & Hu 2012 [68]	23-76 (44.3)	45 + 46	Both (SDS,SAS)	Nonselective	Gynecology	-	②+④+⑥	Nurse (training)	A + B + C
Liu 2012 [48]	45-74 (62.3)	40 + 40	Depression (HAMD)	Nonselective	Mixed	-	① + ② + ④ + ⑥	Nurse	A + C
Shi et al. 2012 [92]	21-65 (53.5)	74 + 74	Both (SDS,SAS)	Nonselective	Gynecology	-	② + ③ + ⑥	-	A
Jia2012 [127]	43-77 (55.8)	35 + 32	Both (HAMD,HAMA)	Nonselective	Head/neck	-	② + ③ + ④ + ⑥ + ⑦	-	A + C
Zhang 2012 [97]	34-71 (63.5)	45 + 45	Both (SDS,SAS)	Nonselective	Mixed	Advanced	② + ⑥	-	-
Chen 2012 [51]	18-79 (51)	43 + 44	Both (SDS,SAS)	Nonselective	Gynecology	-	① + ② + ③ + ⑥	-	A + C
Li et al. 2012 [146]	>18 (57.2)	30 + 30	Both (HAMD,HAMA)	Nonselective	Head/neck	-	① + ② + ③ + ⑦	Doctor (training)/Psychologist	A
Yang & Wang 2012 [83]	29-69	30 + 30	Both (HAMD,HAMA)	Nonselective	Mixed	Advanced	② + ③ + ④ + ⑤ + ⑥	-	A + C
Jiang et al. 2012 [49]	>16	44 + 45	Depression (SDS)	Nonselective	Head/neck	-	① + ② + ③	Nurse	A
Fan & Pan 2012 [158]	30-48	19 + 19	Anxiety (SAS)	Selective	Gynecology	-	② + ④ + ⑥ + ⑦	-	A + C
Li et al. 2012 [72]	24.5-70	50 + 50	Both (SDS,SAS)	Nonselective	Breast	-	② + ③ + ④	Nurse	A + B + C*
Han et al. 2012 [85]	18-91 (74)	43 + 42	Both (SDS,SAS)	Nonselective	Mixed	-	② + ③ + ④ + ⑥	Doctor/Nurse (training)	A + C
Zheng et al. 2012 [175]	25-69 (46.5)	30 + 30	Anxiety (SAS)	Nonselective	Mixed	-	① + ② + ③ + ④ + ⑥	-	A + C
Yuan & Wu 2013 [147]	50-70 (63)	78 + 78	Both (SDS,SAS)	Nonselective	Mixed	Advanced	① + ⑥	-	A + C
Zhu et al. 2013 [36]	43-78	25 + 25	Both (SDS,SAS)	Nonselective	Lung	-	⑦	-	-
Du 2013 [74]	24-76 (46.3)	36 + 36	Both (SDS,SAS)	Nonselective	Gynecology	-	① + ② + ⑥	Nurse/Community Doctor	A + B + C*
Mu et al. 2012 [178]	32-70 (56.2)	60 + 60	Anxiety (SAS)	Nonselective	Urinary	-	① + ② + ③ + ④ + ⑥	Nurse	A + B + C
Liu & Gan 2013 [43]	18-67 (49.3)	101 + 90	Both (SDS,STAI)	Nonselective	Mixed	-	⑤	-	A
Zhang 2013 [96]	32-73	72 + 72	Both (SDS,SAS)	Nonselective	Mixed	Advanced	① + ② + ③ + ④ + ⑥	-	A + C
Zhang et al. 2013 [142]	32-72 (54)	33 + 35	Both (SDS,SAS)	Nonselective	Mixed	-	② + ④ + ⑦	Doctor (training)	B
Guo et al. 2013 [179]	>18 (47)	89 + 89	Both (SDS,SAS)	Nonselective	Mixed	-	① + ②+ ⑦	Clinician/Nurse/Radiation therapist (training)	B
Liu 2013 [119]	31-65 (53.3)	45 + 45	Both (SDS,SAS)	Selective	Mixed	-	③ + ⑥	Nurse	A
Zhai et al. 2013 [82]	47-62 (52)	39 + 39	Both (SDS,SAS)	Nonselective	Head/neck	-	② + ③ + ④ + ⑥	Nurse	A + C
Ci et al. 2013 [117]	25-65	30 + 30	Both (SDS,SAS)	Nonselective	Mixed	-	② + ⑥	Nurse	A + C
Liu 2013 [112]	46-71 (51.4)	59 + 59	Both (SDS,SAS)	Selective	Mixed	-	② + ④	-	A + C
Liu et al. 2013 [57]	35-76 (53)	29 + 29	Both (SDS,SAS)	Nonselective	Bone metastatic	-	① + ② + ③ + ④+ ⑥	Nurse	-
Qiu et al. 2013 [181]	31-64 (50.6)	29 + 25	Both (HAMD,SAS)	Selective	Breast	Early	①	Psychiatrist (CBT and group therapy training)	B
Mao et al. 2013 [80]	>16 (58.2)	100 + 100	Both (SDS,SAS)	Nonselective	Mixed	-	① + ② + ③ + ④ + ⑥	Psychologist	A + C
Zhang 2013 [114]	18-70 (46)	53 + 53	Both (SDS,SAS)	Nonselective	Gynecology	-	① + ② + ③ + ④ + ⑥	Nurse	A + C
Yu 2013 [137]	>18	79 + 41	Both (SDS,SAS)	Nonselective	Head/neck	-	③ + ④ + ⑥	Doctor	A + B + C
Wang 2013 [53]	21-70 (45)	50 + 50	Both (SDS,SAS)	Nonselective	Gynecology	-	① + ②+ ④	Nurse	A + C
Tian et al. 2013 [168]	>18 (61.1)	98 + 97	Anxiety (SAS)	Nonselective	Mixed	-	② + ④ + ⑤ + ⑥	Nurse	A + C
Yu 2013 [172]	33-61 (41.9)	83 + 83	Anxiety (SAS)	Nonselective	Breast	-	② + ④ + ⑥	-	A + C*

*Abbreviations*: *n1* participants in experimental group, *n2* participants in control group, *SDS* Self-rating Depression Scale, *SAS* Self-rating Anxiety Scale, *HAMD* Hamilton Depression Rating Scale, *HAMA* Hamilton Anxiety Rating Scale, *STAI* State-Trait Anxiety Inventory, *DSI* Depression Screening Instrument, *CES-D* Center for Epidemiologic Studies Depression Scale, *SAI* State Anxiety Inventory, *HASD* Hospital Anxiety and Depression Scale, *①* cognitive-behavioral interventions, *②* patients education, *③* relaxation/imagery, *④* social/family support, *⑤* music therapy, *⑥* nursing intervention, *⑦* other interventions, *A* individual, *B* Group, *C* Family, C* Couple, *−* no report.

### Risk of bias assessment

Ratings of study quality for each criteria of the modified Jadad were presented in Table 2. As shown in Table 2, higher scores reflected the better study quality, and the average scores of all studies were above 2 (mean: 2.68). Nineteen studies were judged to have low quality for random sampling or withdrawals/dropouts or inclusion/exclusion criteria or the statistical analysis and twenty-seven of high quality. Other studies were rated as medium quality.

**Table 2:** **Assessment of study quality**

Studies	Quality Indicators from the modified Jadad scale					Total score
	A	B	C	D	E	
Wang et al. 2000 [39]	1	0	0	0	0	1
Zhao et al. 2000 [153]	1	0	0	1	1	3
Cai et al. 2001 [144]	1	0	0	0	1	2
Yang et al. 2002 [42]	1	1	0	1	0	3
Guan et al. 2002 [123]	1	0	0	0	1	2
Li et al. 2002 [76]	1	−1	0	1	1	2
Lian et al. 2003 [44]	1	0	0	1	1	3
Wu & Wang 2003 [148]	1	1	0	1	1	4
Zhong et al. 2003 [38]	1	0	0	0	1	2
Lou et al. 2003 [101]	1	−1	0	1	1	2
Xu 2004 [115]	1	0	0	0	1	2
Wang 2004 [93]	1	0	0	0	0	1
Bu et al. 2005 [155]	1	1	1	1	1	5
Lou et al. 2005 [164]	1	−1	0	0	1	1
Liu et al. 2006 [143]	1	0	1	0	1	3
Cheng et al. 2006 [107]	1	0	0	0	0	1
Wang et al. 2006 [75]	1	0	1	1	0	3
Ni et al. 2007 [165]	1	0	0	0	1	2
Pang & Wang 2007 [166]	1	0	0	0	1	2
Qian & Cai 2007 [50]	1	1	0	1	1	4
Wen & Liang 2007 [69]	1	0	0	1	0	2
Kang 2007 [128]	1	0	0	0	0	1
Zheng et al. 2007 [109]	1	0	0	1	1	3
Deng et al. 2007 [110]	1	0	0	1	1	3
Xing 2007 [103]	1	0	0	1	1	3
Wu et al. 2007 [59]	1	0	0	1	1	3
Xu 2007 [88]	1	0	0	1	0	2
Han & Liu 2007 [151]	1	−1	0	0	1	1
Huang et al. 2008 [54]	1	0	0	1	0	2
Zheng et al. 2008 [116]	1	0	0	1	1	3
Yang 2008 [79]	1	1	0	1	0	3
Han 2008 [160]	1	0	0	0	1	2
Jiang et al. 2008 [161]	1	0	0	1	1	3
Li et al. 2008 [163]	1	0	0	0	1	2
Wang et al. 2008 [169]	1	0	0	1	1	3
Ji 2008 [106]	1	−1	0	0	1	1
Jin & Zhu 2008 [122]	1	0	0	0	0	1
Li et al. 2008 [99]	1	0	0	0	1	2
Liu et al. 2008 [52]	1	1	0	0	1	3
Yang 2008 [136]	1	1	0	1	1	4
Zhou 2008 [100]	1	0	0	0	0	1
Mao et al. 2008 [113]	1	1	0	0	1	3
Liu 2008 [132]	1	1	0	1	1	4
Zheng et al. 2008 [125]	1	0	0	1	1	3
Chen et al. 2009 [156]	1	0	0	0	1	2
Li 2009 [89]	1	0	0	0	1	2
Li et al. 2009 [78]	1	0	0	1	1	3
Fu et al. 2009 [145]	1	−1	0	0	1	1
Qiu 2009 [133]	1	0	0	1	1	3
Sun 2009 [134]	1	0	0	1	1	3
Xia 2009 [118]	1	−1	0	1	1	2
Zhang 2009 [139]	1	1	1	1	1	5
Zhou 2009 [140]	1	0	0	1	1	3
Li et al. 2009 [63]	1	−1	0	0	1	1
Geng et al. 2010 [104]	1	0	1	1	1	4
Zhan & Cheng 2010 [105]	1	1	0	1	1	4
Cheng et al. 2010 [45]	1	0	0	1	1	3
Li et al. 2010 [91]	1	1	0	0	1	3
Guan et al. 2010 [81]	1	0	0	1	1	3
Li 2010 [111]	1	−1	0	1	1	2
Zhang 2010 [138]	1	1	1	1	1	5
Su & Wang 2010 [167]	1	1	1	1	1	5
Fu et al. 2010 [159]	1	0	0	0	1	2
Wu & Zhang 2010 [170]	1	0	0	0	1	2
Zhou 2010 [176]	1	−1	0	0	1	1
You et al. 2010 [171]	1	0	0	1	1	3
Ren et al. 2010 [154]	1	1	0	1	1	4
Xu 2010 [40]	1	0	0	1	1	3
Guo et al. 2010 [71]	1	0	1	1	1	4
Tang et al. 2010 [126]	1	−1	0	1	1	2
Liu et al. 2010 [46]	1	0	0	0	1	2
Shi et al. 2010 [108]	1	0	0	1	1	3
Liu et al. 2010 [87]	1	1	0	1	1	4
Wang 2010 [90]	1	−1	0	1	1	2
Huang et al. 2010 [86]	1	0	0	0	1	2
Zhang & Yu 2011 [94]	1	−1	0	0	0	0
Du et al. 2011 [61]	1	0	0	1	1	3
Li et al. 2011 [149]	1	1	0	1	1	4
Zhou et al. 2011 [180]	1	1	1	1	1	5
Liu 2011 [131]	1	0	0	1	1	3
Shen et al. 2011 [64]	1	0	1	1	1	4
Zhu et al. 2011 [65]	1	−1	0	1	1	2
Meng et al. 2011 [95]	1	0	0	0	1	2
Dai et al. 2011 [157]	1	−1	0	1	1	2
Jiao et al. 2011 [162]	1	1	0	0	1	3
Ye 2011 [56]	1	0	0	0	1	2
Li2011 [130]	1	1	0	1	1	4
Liu et al. 2011 [41]	1	0	0	1	1	3
Wang et al. 2011 [37]	1	−1	1	1	0	2
Cao 2011 [173]	1	−1	0	1	1	2
Zhao & Zhang 2011 [174]	1	0	0	1	1	3
Cao & Li 2011 [67]	1	0	0	1	1	3
Huang et al. 2011 [152]	1	0	0	0	1	2
Hu & Yan 2011 [62]	1	1	1	1	1	5
Guan & Jin 2011 [120]	1	1	0	1	1	4
Lv et al. 2011 [77]	1	−1	0	0	1	1
Li et al. 2011 [182]	1	1	1	1	1	5
Cao & Jiang 2011 [177]	1	0	0	1	1	3
Huang 2011 [102]	1	0	0	1	1	3
Zheng et al. 2011 [141]	1	1	0	1	1	4
Wu & Dong 2011 [47]	1	0	0	1	1	3
Zheng et al. 2012 [150]	1	0	0	1	0	2
Wang & Xiao 2012 [124]	1	0	0	1	1	3
Wei 2012 [135]	1	0	0	1	0	2
Feng 2012 [55]	1	−1	0	0	1	1
Yang et al. 2012 [73]	1	0	0	1	1	3
Zhao et al. 2012 [84]	1	0	0	1	1	3
Gu 2012 [121]	1	0	0	1	1	3
Zheng 2012 [58]	1	−1	0	1	1	2
Yang 2012 [66]	1	0	0	1	1	3
Sun et al. 2012 [60]	1	0	0	1	1	3
Liu et al. 2012 [98]	1	0	0	1	1	3
Yang et al. 2012 [70]	1	0	0	1	1	3
Li 2012 [129]	1	0	1	1	1	4
Zhu & Hu 2012 [68]	1	1	0	1	1	4
Liu 2012 [48]	1	1	0	1	1	4
Shi et al. 2012 [92]	1	1	0	0	1	3
Jia 2012 [127]	1	0	0	1	0	2
Zhang 2012 [97]	1	0	0	0	1	2
Chen 2012 [51]	1	0	0	1	1	3
Li et al. 2012 [146]	1	1	0	0	1	3
Yang & Wang 2012 [83]	1	0	0	1	1	3
Jiang et al. 2012 [49]	1	−1	0	1	1	2
Fan & Pan 2012 [158]	1	−1	0	0	1	1
Li et al. 2012 [72]	1	0	0	1	1	3
Han et al. 2012 [85]	1	0	0	1	1	3
Zheng et al. 2012 [175]	1	0	0	0	1	2
Yuan & Wu 2013 [147]	1	0	0	1	1	3
Zhu et al. 2013 [36]	1	−1	0	1	1	2
Du 2013 [74]	1	0	0	0	0	1
Mu et al. 2012 [178]	1	0	0	0	1	2
Liu & Gan 2013 [43]	1	−1	0	1	1	2
Zhang 2013 [96]	1	0	0	1	1	3
Zhang et al. 2013 [142]	1	1	0	0	1	3
Guo et al. 2013 [179]	1	1	1	1	1	5
Liu 2013 [119]	1	0	0	0	1	2
Zhai et al. 2013 [82]	1	0	0	1	1	3
Ci et al. 2013 [117]	1	0	0	0	1	2
Liu 2013 [112]	1	0	0	0	0	1
Liu et al. 2013 [57]	1	0	0	1	1	3
Qiu et al. 2013 [181]	1	1	1	1	1	5
Mao et al. 2013 [80]	1	0	0	1	1	3
Zhang 2013 [114]	1	0	0	1	1	3
Yu 2013 [137]	1	0	0	1	1	3
Wang 2013 [53]	1	0	0	0	1	2
Tian et al. 2013 [168]	1	0	1	1	1	4
Yu 2013 [172]	1	−1	0	0	1	1

Note: The modified Jadad scale is an eight-item scale. Considering the characteristic and effect of psychological interventions, blinding (2 points) and adverse effects (1 point) were excluded.

*Abbreviations*: A represents “Was the study described as randomized?” (1: Yes; 0: No); B represents “Was the method of randomization appropriate?” (1: Yes; 0: Not described; −1: No); C represents “Was there a description of withdrawals and dropouts?” (1: Yes; 0: No); D represents “Was there a clear description of the inclusion/exclusion criteria?” (1: Yes; 0: No); E represents “Was the methods of statistical analysis described?” (1: Yes; 0: No).

### Effects of psychological interventions on depression and anxiety in cancer patients

**Figure 3:**
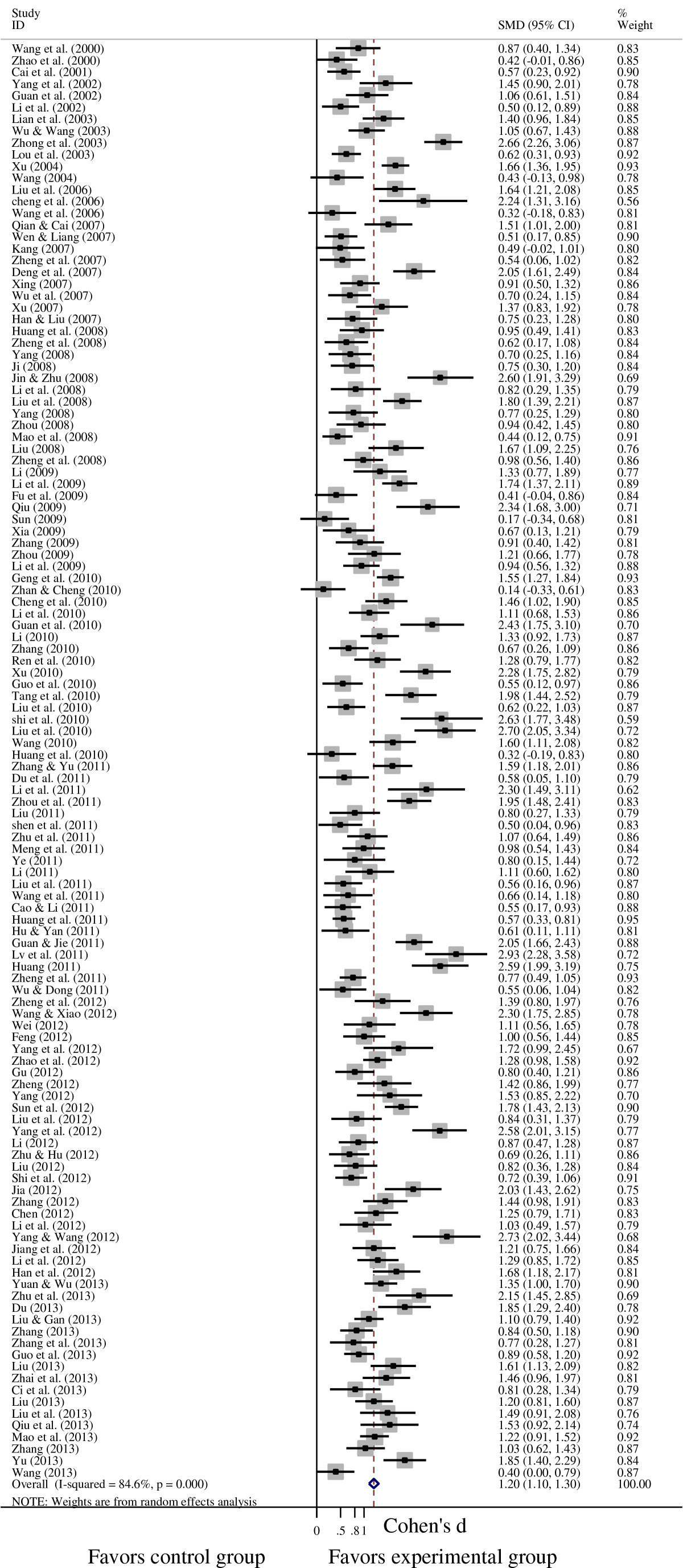
**Forest plot of the effects of psychological interventions on depression in cancer patients.** It shows a pooled SMD of 1.199 (95% CI = 1.095-1.303; p < 0.001), indicating that psychological interventions could alleviate depression among Chinese adults with cancer. Abbreviations: SMD, standardized mean difference.

**Figure 4:**
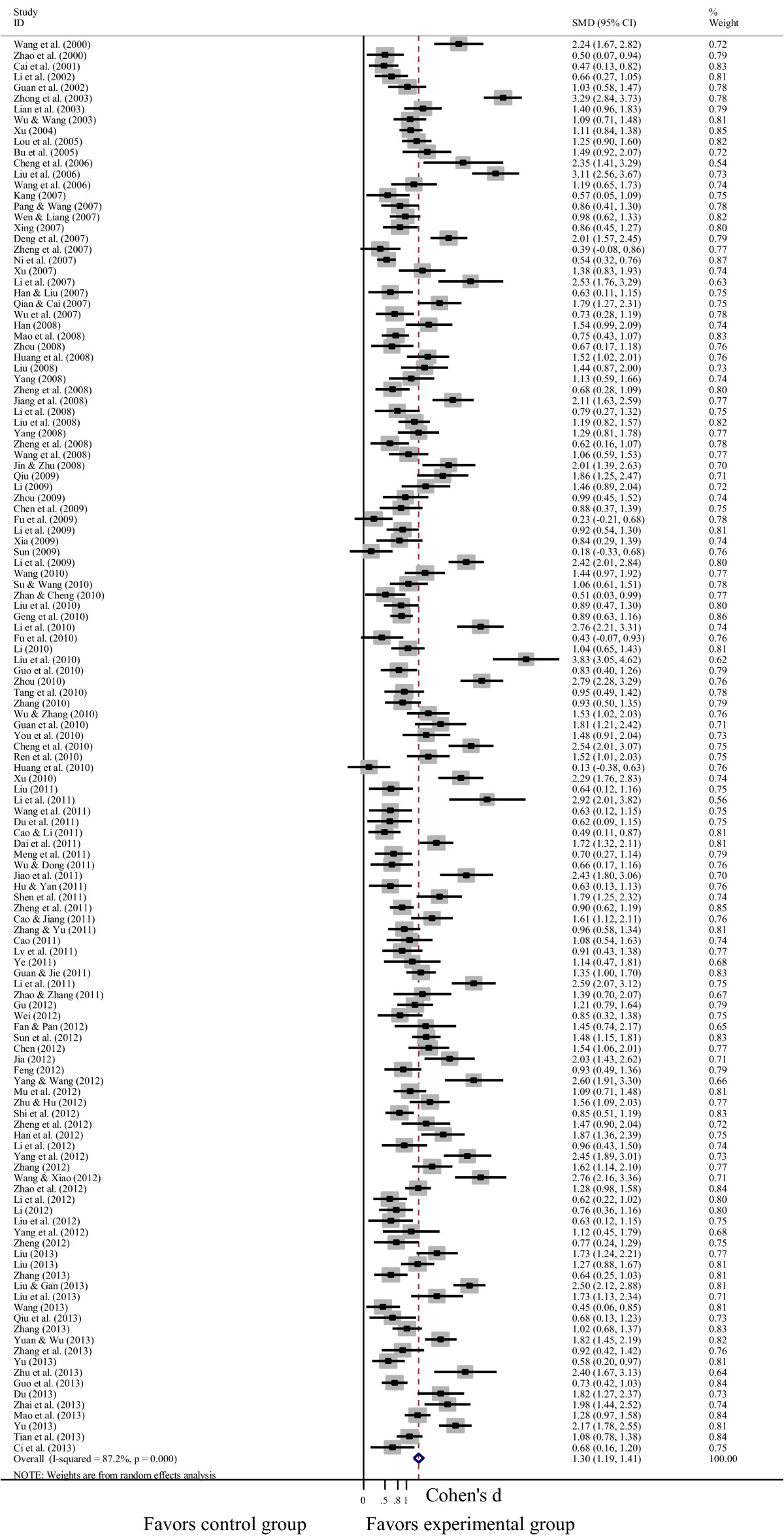
**Forest plot of the effects of psychological interventions on anxiety in cancer patients.** It shows a pooled SMD of 1.298 (95% CI = 1.187-1.408; p < 0.001), indicating that psychological interventions could alleviate anxiety among Chinese adults with cancer. Abbreviations: SMD, standardized mean difference.

A pooled random-effects meta-analysis was conducted using data from 147 studies, which estimated the post-test effects of psychological interventions on depression and anxiety compared with care-as-usual control group. This meta-analysis included data for 7,181 patients in the experimental group, and 6,858 patients in the control group. As shown in Figures 3 and 4, the random effects model showed an overall effect size of SMD = 1.199 (95% CI = 1.095-1.303; p < 0.001) for depression in 122 studies, and a large effect size was also observed (SMD = 1.298, 95% CI = 1.187-1.408; p < 0.001) for anxiety in 131 studies. However, the heterogeneity analysis of the effect sizes of depression (Q = 787.21, p < 0.001; I^2^ = 84.6%) and anxiety (Q = 1016.74, p < 0.001; I^2^ = 87.2%) indicated that there was a relatively high amount of heterogeneity in our meta-analysis.

### Moderator analysis

In univariate and multiple meta-regressions analysis (in Additional files 1 and 2), no moderating effects were found for patients’ age, simple size, intervention type and quality of study (p > 0.05). As shown in Table 3, within the subgroup of studies evaluating moderator variables, significant effects of cancer type were found for depression (p < 0.001) and anxiety (p = 0.02). Effect size in patients with lung cancer was the largest (Depression: SMD = 1.481, 95% CI = 0.811-2.151; Anxiety: SMD = 1.588, 95% CI = 0.994-2.182), but among patients with breast patients, it was the smallest (Depression: SMD = 1.106, 95% CI = 0.830-1.382; Anxiety: SMD = 1.153, 95% CI = 0.857-1.448). Compared with the unselected patients (SMD = 1.170, 95% CI = 1.058-1.282), the effects of psychological interventions on depression were larger (SMD = 1.368, 95% CI = 1.095-1.642) in cancer patients with clear signs of depression/anxiety. Individual psychotherapy (SMD = 1.575, 95% CI = 1.266-1.884) showed a larger effect size on anxiety than the other intervention formats did (SMD = 1.161, 95% CI = 1.045-1.276), and the effect size was the largest in the studies using the State-Trait Anxiety Inventory (STAI) to assess anxiety among cancer patients (SMD = 1.800, 95% CI = 0.717-2.884).

**Table 3:** **Effects of psychological interventions on depression and anxiety in adult with cancer: subgroup analyses**

Subgroup	No. of studies	No. of subjects	SMD	95% CI	Q	I^2^(%)	P^a^
**Depression**							
Caner type^b^							<0.001
Mixed cancer	60	6506	1.113	0.966-1.260	440.61***	86.6	
Lung cancer	8	592	1.481	0.811-2.151	90.26***	92.2	
Head/neck cancer	8	734	1.403	1.150-1.167	16.50*	57.6	
Gynecological cancer	19	1592	1.268	1.015-1.520	94.81***	81.0	
Breast cancer	15	1114	1.106	0.830-1.382	63.82***	78.1	
Digestive tract cancer	9	851	1.283	0.928-1.638	40.26***	80.1	
Cancer stage							0.502
Advanced	21	1810	1.220	0.927-1.513	160.64***	87.5	
Early	8	596	1.401	0.821-1.980	70.28***	90.0	
Patients’ selection							0.004
Nonselective	103	10310	1.170	1.058-1.282	693.41***	85.3	
Selective	19	1261	1.368	1.095-1.642	85.42***	78.9	
Intervention format							0.202
Individual	25	2103	1.256	1.015-1.497	151.98***	84.2	
Other formats	79	7811	1.167	1.043-1.291	490.91***	84.1	
Appropriate randomization							0.923
No	19	1788	1.161	0.920-1.401	98.77***	81.8	
Yes	27	2572	1.145	0.990-1.300	152.60***	83.0	
Questionnaires							1.000
SDS	104	10134	1.189	1.080-1.298	646.84***	84.1	
HAMD	15	1104	1.442	1.050-1.834	114.76***	87.8	
Timing of assessment^c^							0.113
≤1 week	8	759	1.180	0.698-1.662	63.77***	89.0	
2 weeks-4 weeks	22	2599	1.150	0.934-1.366	134.10***	84.3	
6 weeks-8 weeks	19	1644	1.226	0.940-1.512	124.72***	85.6	
>8 weeks	9	765	1.323	0.922-1.724	49.61***	83.9	
**Anxiety**							
Caner type^b^							0.020
Mixed cancer	58	6563	1.242	1.075-1.409	538.86***	89.4	
Lung cancer	9	676	1.588	0.994-2.182	90.75***	91.2	
Head/neck cancer	7	645	1.468	0.943-1.992	51.74***	88.4	
Gynecological cancer	22	1740	1.385	1.139-1.630	110.22***	80.9	
Breast cancer	20	1622	1.153	0.857-1.448	141.59***	86.6	
Digestive tract cancer	11	1020	1.371	1.024-1.718	58.7***	83.0	
Cancer stage							0.777
Advanced	22	2175	1.178	0.923-1.434	154.64***	86.4	
Early	7	512	1.271	0.687-1.855	54.27***	88.9	
Patients’ selection							0.114
Nonselective	111	11241	1.322	1.201-1.444	932.67***	88.2	
Selective	20	1327	1.152	0.906-1.399	81.57***	76.7	
Intervention format							<0.001
Individual	28	2287	1.575	1.266-1.884	277.89***	90.3	
Other formats	81	8285	1.161	1.045-1.276	464.52***	82.8	
Appropriate randomization							0.458
No	21	2018	1.245	0.955-1.535	172.30***	88.4	
Yes	26	2508	1.383	1.140-1.627	187.05***	86.6	
Questionnaires							<0.001
SAS	113	10918	1.276	1.163-1.390	810.72***	86.2	
HAMA	9	624	1.295	0.856-1.733	48.06***	83.4	
STAI	4	546	1.800	0.717-2.884	83.95***	96.4	
SAI	4	420	1.639	0.916-2.362	30.86***	90.3	
Timing of assessment^c^							0.246
≤1 week	10	1211	1.224	0.881-1.566	64.58***	86.1	
2 weeks-4 weeks	24	2519	1.207	0.986-1.427	145.26***	84.2	
6 weeks-8 weeks	14	1294	1.283	0.920-1.646	114.14***	88.6	
>8 weeks	8	658	1.021	0.801-1.241	12.10	42.2	

*Abbreviations*: *SDS* Self-rating Depression Scale, *SAS* Self-rating Anxiety Scale, *HAMD* Hamilton Depression Rating Scale, *HAMA* Hamilton Anxiety Rating Scale, *STAI* State-Trait Anxiety Inventory, *SAI* State Anxiety Inventory.

*p < 0.05.

***p < 0.001.

^a^P of comparison between these subgroups [30], which is akin to analysis of variance. We partition the total variance into variance within groups and variance between groups, and then test these various components of variance for statistical significance, with the last (variance between groups) addressing the hypothesis that effect size differs as function of group membership.

^b^Due to a few of studies (the number is less than or equal to 2) separately reporting the effect size for depression and anxiety in patients with urinary cancer, bone metastatic cancer, and blood cancer, the subgroup comparison of depression and anxiety in these cancer types were not included.

^c^Timing of assessment was aimed at the specified time range (e.g., days, weeks, months and years) post-treatment(e.g., surgery and chemotherapy) or post-intervention.

### Publication bias

Visual inspection of the funnel plot indicated some publication bias, and the Begg’s test and Egger’s test further suggested the publication bias in depression (Begg’s test, Z = 4.16, P < 0.001; Egger’s test, Coef = 3.659, P < 0.001) and anxiety (Begg’s test, Z = 4.99, P < 0.001; Egger’s test, Coef = 4.469, P < 0.001) in our meta-analysis.

### Cumulative meta-analysis

**Figure 5:**
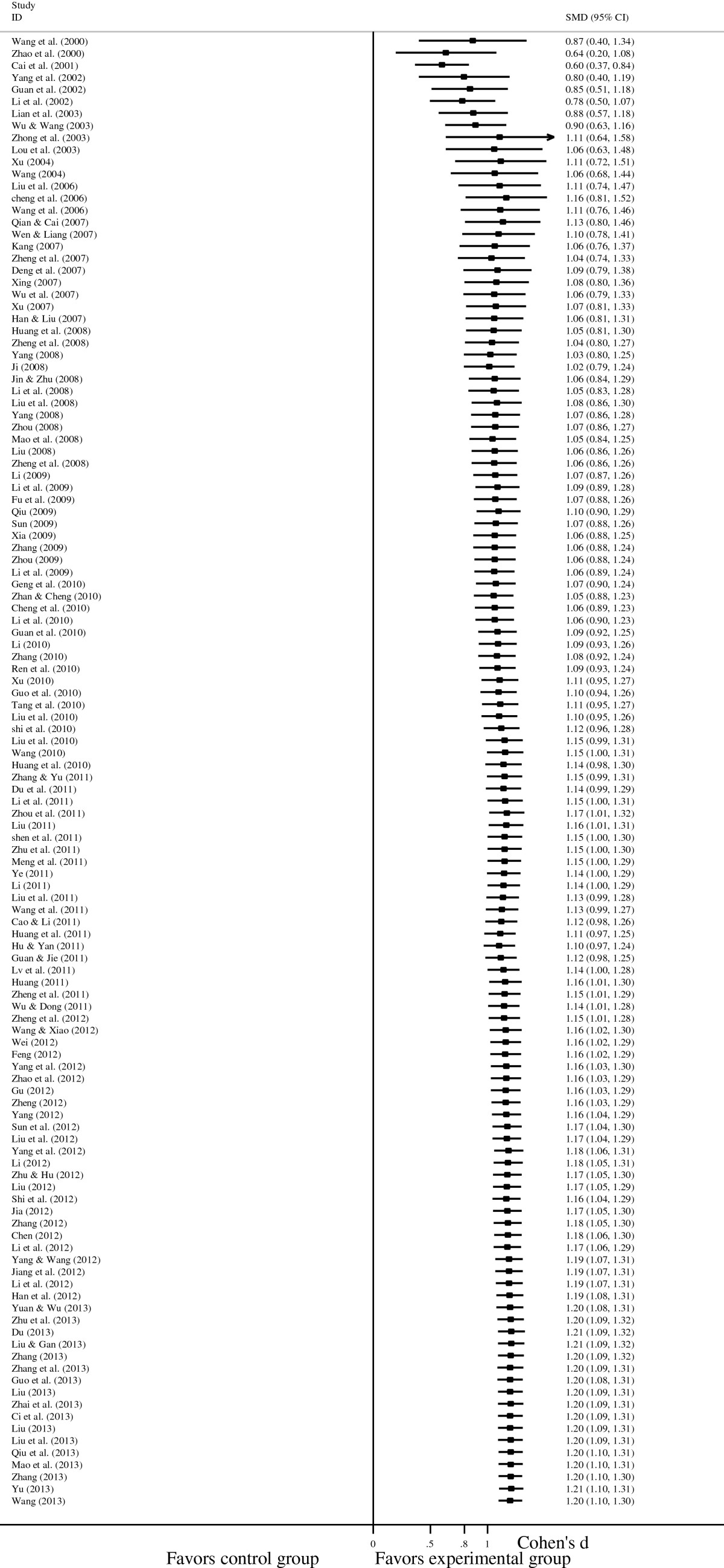
**Cumulative meta-analysis of randomized trials comparing psychological interventions with control: depression.** Abbreviations: SMD, standardized mean difference.

**Figure 6:**
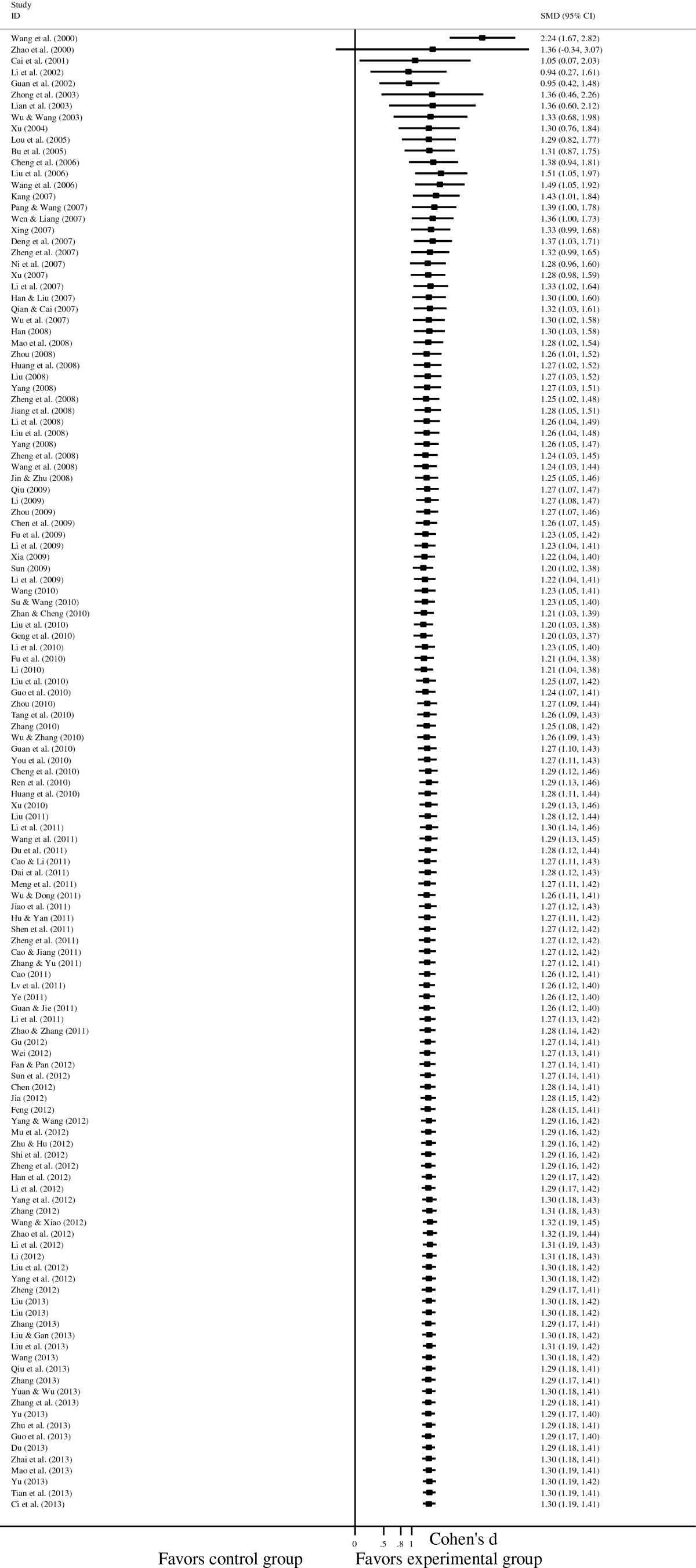
**Cumulative meta-analysis of randomized trials comparing psychological interventions with control: anxiety.** Abbreviations: SMD, standardized mean difference.

Cumulative meta-analysis (Figure 5) indicated that the protective effects of psychological interventions on depression became evident in 2000. Since 2012, the overall effect size (SMD) has remained relatively stable (range: 1.15 - 1.21), and subsequent studies published in 2013 hardly changed the overall effect size. The protective effects of psychological interventions on anxiety became evident in 2001 (Figure 6). Sufficient body of RCTs had accumulated by 2003 to determine a reliable and consistent point estimate (fluctuated around 1.3), and resulted in a narrowing of the 95% CI.

## Discussion

At the beginning of discussion, we would evaluate the heterogeneity and study quality in the present meta-analysis. First, we performed strict inclusion criteria, random effects models and moderator analysis to control and reduce the heterogeneity. However, the heterogeneity was still relatively high, and the conclusion should be considered with some caution. Second, the modified Jadad scale was used to assess the study quality. Although most of the included studies (87%) had medium-quality or high-quality, studies in our meta-analysis had the high bias of the inappropriate methods of randomization (79%) and the lack of description of withdrawals/dropouts (88%). Quality assessment indicated these methodological weaknesses, which could weaken the internal validity.

In the present meta-analysis, we analyzed the effects of psychological interventions on depression and anxiety among Chinese adults with cancer. To our knowledge, this is the largest and the most comprehensive meta-analysis studying the effects of psychological interventions on psychological distress in Chinese adults with cancer, and psychological interventions were proven effective to relieve cancer patients’ depression (SMD = 1.199, 95% CI = 1.095-1.303) and anxiety (SMD = 1.298, 95% CI = 1.187-1.408) in our meta-analysis. Although the research of psychological interventions in cancer patients is quite common, the large and comprehensive meta-analysis conducted by foreign researchers usually excluded Chinese studies because they were published in a foreign language [20]. Some Chinese meta-analysis in this field only included only a small number of studies (n = 11) [183, 184], which did not accurately reflect the current research of psychological interventions in Chinese cancer patients. On the other hand, the results of cumulative meta-analysis showed that the protective effects of psychological interventions on depression/anxiety were evident from 2000–2001 onwards. Subsequent included studies have only tried to increase the precision and reliability of effectiveness of psychological interventions in Chinese adults with cancer, and the overall effect size was both substantial and unlikely to be changed by the further RCTs evidence.

We also compared our results with three other relatively comprehensive meta-analyses exploring the effects of psychological interventions on depression/anxiety in cancer patients: (1) the study of psycho-oncologic interventions on emotional distress and quality of life in adult patients with cancer conducted by Faller (Depression: n = 72, Cohen’s d = 0.33, 95% CI = 0.25-0.41; Anxiety: n = 74, Cohen’s d = 0.38, 95% CI = 0.29-0.46) [20]; (2) the research of the effects of psychological interventions on anxiety/depression in cancer patients reported by Sheard (Depression: n = 20, effect size = 0.36, 95% CI = 0.06-0.66; Anxiety: n = 19, effect size = 0.42, 95% CI = 0.08-0.74) [19]; (3) the review of psychosocial interventions to improve quality of life and emotional wellbeing for recently diagnosed cancer patients conducted by Galway (Depression: n = 6, SMD = 0.12, 95% CI = −0.07-0.31; Anxiety: n = 4, SMD = 0.05, 95% CI = −0.13-0.22) [17]. There might be several reasons for the different effect sizes. The first explanation might be that nearly half of the included studies in our meta-analysis (48%) adopted the psychosocial interventions targeting at both patient and their family members (this percentage in Faller’s study [20] is 4%). Psychosocial interventions involving family members have been proven to be beneficial for depression of chronic illness patients, including cancer patients [185]. Moreover, family is the bedrock of Chinese society, and the care and concern of family members are of great importance for cancer patients. Second, most of the included studies of these meta-analyses are from developed countries and have lower prevalence of mental health problems as compared to developing countries like China [186], and our previous studies also found that the prevalence of depression/anxiety was very high among Chinese cancer patients [10, 187]. Psychological interventions on depression and anxiety were generally highly effective when psychological distress was at a high level at baseline [188]. Third, with the exception of one study of outpatients in non-hospital setting [74], all of the patients in our study were inpatients who had adequate time and appropriate locations to receive psychological interventions (this percentage of inpatients in Faller’s study [20] is 16%), thus with a reduced risk for drop-out. Studies on the issues of compliance/dropout claimed that drop-out rate was an important indicator of therapeutic effectiveness [189]. Therefore, the large effect size in our study may be due to the reduced risk of drop-out. The last explanation might be that these three meta-analyses mainly included breast cancer patients (Faller’s study: 39% [20]; Galway’s study: 30% [17]). Our meta-analysis found that the effect of psychological interventions on depression/anxiety was the smallest among breast cancer patients (this percentage in our study was 15%), and this might also inflate the overall effect sizes.

In the present meta-analysis, no moderating effect was found for intervention type (continuous variable) in univariate and multiple meta-regressions analysis. Similar to the results of our meta-analysis, most of psychological interventions in other comprehensive meta-analyses were integrative [17, 19, 20], so it could be difficult to compare the effects of different psychotherapies. However, significant medium-to-large effects were observed for the meta-analyses focusing on the separate psychological interventions (e.g., mindfulness-based therapy, relaxation/imagery, and CBT) [21–23], indicating that the quality and content of psychological interventions could be more important for cancer patients than the total types of included interventions.

Through the subgroups analysis of moderator variables (categorical variable), significant moderator effects were found for cancer type, patients’ selection, intervention format and questionnaires employed. The order of the effects of psychological interventions on depression/anxiety was lung cancer, head/neck cancer, digestive tract/gynecological cancer and breast cancer among different types of cancer. The epidemiological features and the psychosocial problems of the specific types of cancer might be the leading cause of this result. Lung cancer is the leading cause of cancer death in both men and women in China and the world [24, 190], and lung cancer patients were at higher risk for psychosocial problems (e.g., stigma and depression) [191, 192]. For example, lung cancer patients experienced the greatest amount of psychological distress among 14 types of cancer [193]. Head/neck and gynecological cancer patients also experienced the unique stress and psychological problems. Patients had to face stigma, functional impairment and disfigurement caused by the cancer and/or the treatment [191, 194], and gynecological cancer patients had problems including stigma, self-image, female fertility, and changes in sexual function [187, 191]. However, in China, the death rate of breast cancer was at a medium or low level (ranked as the fifth following lung cancer and digestive tract cancer) [190], and survival rates have increased to the extent that more than 70% now survived 5 years after diagnosis in urban areas [195]. In a large cancer cohort, higher rates of mixed anxiety/depression symptoms were found in patients with digestive tract, head/neck, and lung cancers, while lower rates were observed in those with breast cancers [196]. As a result, compared with breast cancer patients, other types of cancer patients might have a higher level of psychological distress, and the effects of psychological interventions on depression/anxiety were larger in patients with lung, head/neck, digestive tract, and gynecological cancers [188].

Effect sizes of patients with clear signs of depression/anxiety were significantly larger for depression, and individually based interventions were more effective for anxiety than those delivered in other formats. Psychological interventions appeared to be more useful for patients with increased psychological distress, which was similar to the findings of other meta-analyses in this field [17, 19, 20], indicating that compared with non-screened patients, patients with clear signs of psychological distress could benefit more from psychological interventions, and the effects of interventions targeted at those at risk of psychological distress would be much larger. Additionally, individual interventions appeared to be more effective for anxiety in our meta-analysis, indicating that individual therapy could be more suitable for anxiety among cancer patients. Individual interventions were better suited to handle particular, individual and internal problems [197], and to some extent, anxiety is a normal and individual reaction when a person is faced with different stressors, including cancer [10, 187]. Therefore, individual interventions might be more helpful to deal with the anxiety caused by different types of cancer. However, some studies reported the conflicting results [19, 21, 198], and more studies are needed to confirm whether the effects of psychotherapy on psychological distress are affected by intervention format. Finally, in addition to considering these moderator effects, it is also important to evaluate the influence of different kinds of questionnaires employed on the outcomes of psychological interventions among cancer patients.

### Implication

There are several theoretical and practical implications for our meta-analysis. In theory, although cultural traditions, life experience, social economy and ideology were different between China and Western countries, the present meta-analysis suggested that the psychological interventions (or psychotherapies) widely used in Western countries are also suitable and even more efficacious in Eastern culture context. In practice, first, some developed countries, such as United States and Australia, have developed several clinical practice guidelines for the psychotherapy and supportive care of cancer patients [199], but the corresponding management systems and processes are still not available in China. Therefore, the Chinese government and Chinese medical settings should set up an adequate institutional and organizational system to provide routine use of psychological interventions in cancer patients; second, when interventions are performed, quality and content of interventions might be more important for cancer patients than the total types of included interventions, and further studies should be conducted to explore whether psychological interventions involving family members will be more effective for depression/anxiety in cancer patients; third, our findings also provided guidance in developing optimal methods and appropriate standards of psychological interventions in clinical practice. For example, oncologists and physicians should pay more attention to detecting depression/anxiety of specific types of cancer (e.g., lung cancer), and necessary and timely psychological interventions should be taken to alleviate depression/anxiety in these cancer patients. Moreover, psychotherapeutic programs should screen and preselect patients with clear signs of depression/anxiety, so that the limited clinical resources in China could be appropriately allocated and produce maximal cost-effectiveness and clinical benefits.

### Limitation

The present meta-analysis had several limitations. First, our meta-analysis did not provide enough information and number of studies regarding other potential moderating factors, such as gender, income, intervention sessions and duration, and metastasis. Second, although we employed moderator analysis to explore potential sources of heterogeneity, the moderator analysis could not reduce I^2^ to 75% or less in many cases. This may be mainly because interventions in our meta-analysis varied greatly with respect to intervention type and professionalism of therapists, and other important moderating factors. Third, most of the included studies were conducted using self-rating questionnaires (e.g., SAS and SDS) to measure depression and anxiety. Therefore, depression and anxiety in our meta-analysis more often referred to the depressive symptom and anxiety symptom. Fourth, because follow-up results after post-test were not reported, it is not confirmed whether there were long term effects. Fifth, unpublished researches were not included in our meta-analysis, and unpublished outcomes were often insignificant, which might inflate the effect sizes in the presented study. Finally, the high risk of publication bias is another (and perhaps the most important) limitation. This might be mainly because unlike some foreign medical journals that require registration of a trial before it commences, the systems related to registries have not yet been established in China. Thus, attempts to identify unpublished studies are very difficult.

## Conclusions

Although there are some clear limitations (heterogeneity and publication bias) in this study, a tentative and preliminary conclusion can be reached, that psychological interventions of depression and anxiety are effective for Chinese cancer patients. In studies that included lung cancer, preselected patients with clear signs of depression/anxiety, adopted individual intervention and used STAI, the effect sizes are larger. The findings support that an adequate system should be set up to provide routine psychological interventions for cancer patients in Chinese medical settings.

## Electronic supplementary material

Additional file 1: Effects of psychological interventions on depression and anxiety in adult with cancer: Univariate and multiple meta-regressions analysis.(DOC 398 KB)

Additional file 2: Table S1: Characteristics of the included studies. (DOC 386 KB)

Below are the links to the authors’ original submitted files for images.

Authors’ original file for figure 1

Authors’ original file for figure 2

Authors’ original file for figure 3

Authors’ original file for figure 4

Authors’ original file for figure 5

Authors’ original file for figure 6
