# Supplementary material for: The effects of psychological interventions on depression and anxiety among Chinese adults with cancer: a meta-analysis of randomized controlled studies
Source: BMC Cancer. 2014 Dec 15;14:956. doi: 10.1186/1471-2407-14-956 (PMC4301929; doi:10.1186/1471-2407-14-956)
Supplement: Supplementary file 1 — Additional file 1: Effects of psychological interventions on depression and anxiety in adult with cancer: Univariate and multiple meta-regressions analysis.(DOC 398 KB) [file 12885_2014_5105_MOESM1_ESM.doc]

**Additional table**

**Effects of psychological interventions on depression and anxiety in adult with cancer: Univariate and multiple meta-regressions analysis**

| **Variables (Univariate)** | Coef. | Std. Err. | t | P value | 95%CI |
| --- | --- | --- | --- | --- | --- |
| Age | 0.0124 | 0.0092 | 1.35 | 0.182 | -0.0059-0.0308 |
| Simple size | -0.003 | 0.0012 | -0.23 | 0.821 | -0.0027-0.0022 |
| Sum types of intervention | 0.0473 | 0.0474 | 1.00 | 0.320 | -0.0465-0.1410 |
| Quality of study | -0.0029 | 0.0558 | -0.05 | 0.959 | -0.1135-0.1077 |
| **Variables (Multivariate)** |  |  |  |  |  |
| Age | 0.0099 | 0.0097 | 1.02 | 0.312 | -0.0094-0.0292 |
| Simple size | -0.002 | 0.0015 | -0.20 | 0.845 | -0.0033-0.0027 |
| Sum types of intervention | 0.0601 | 0.0579 | 1.04 | 0.303 | -0.0551-0.1753 |
| Quality of study | 0.0028 | 0.0640 | 0.04 | 0.965 | -0.5809-1.5815 |

**Table 1 Characteristics of the included studies (supplementary**)

| Author & Years | Timing of assessment | Treatments | Outcomes | Mean1 | SD1 | Mean2 | SD2 |
| --- | --- | --- | --- | --- | --- | --- | --- |
| Wang et al.2000[39] | Post-radiotherapy | Radiotherapy | Depression | 0.71 | 0.25 | 0.52 | 0.18 |
|  |  |  | Anxiety | 59.7 | 11.2 | 36.2 | 9.7 |
| Zhao et al.2000[153] | 2 months post-treatment | - | Depression | 41.23 | 11.29 | 36.56 | 10.81 |
|  |  |  | Anxiety | 41.23 | 11.70 | 35.67 | 10.33 |
| Cai et al.2001[144] | Post-intervention | Chemotherapy /Chinese medicine | Depression | 50.75 | 9.02 | 45.74 | 8.61 |
|  |  |  | Anxiety | 50.16 | 6.52 | 46.38 | 8.46 |
| Yang et al.2002[42] | 8 weeks post-treatment | Chemotherapy/ Radiotherapy | Depression | 21.6 | 5.4 | 14.2 | 4.8 |
| Guan et al.2002[123] | 1 month post-treatment | - | Depression | 58.34 | 6.14 | 52.56 | 4.67 |
|  |  |  | Anxiety | 56.75 | 8.00 | 49.13 | 6.80 |
| Li et al. 2002[76] | 1 month post-surgery | Surgery | Depression | 45.94 | 10.36 | 40.83 | 10.02 |
|  |  |  | Anxiety | 43.52 | 10.11 | 36.90 | 9.98 |
| Lian et al. 2003[44] | Post-radiotherapy | Radiotherapy | Depression | 57.44 | 6.80 | 47.80 | 6.95 |
|  |  |  | Anxiety | 52.25 | 7.90 | 42.25 | 6.82 |
| Wu&Wang 2003[148] | 1 month post-treatment | Radiotherapy/  Chemotherapy | Depression | 42 | 11 | 31 | 10 |
|  |  |  | Anxiety | 44 | 11 | 32 | 11 |
| Zhong et al. 2003[38] | Post-radiotherapy | Radiotherapy | Depression | 49.8 | 6.83 | 34.26 | 4.62 |
|  |  |  | Anxiety | 46.47 | 4.80 | 31.47 | 4.31 |
| Lou et al. 2003[101] | 1 week post-intervention | Chemotherapy | Depression | 47.3 | 12.9 | 39.4 | 12.5 |
| Xu2004[115] | 4 weeks post-intervention | Surgery | Depression | 0.611 | 0.044 | 0.543 | 0.039 |
|  |  |  | Anxiety | 0.581 | 0.040 | 0.531 | 0.048 |
| Wang 2004[93] | 5 days post-intervention | Chemotherapy | Depression | 35.42 | 9.21 | 31.48 | 9.21 |
| Bu et al.2005[158] | 3 weeks post-intervention | - | Anxiety | 49.37 | 6.28 | 40.46 | 5.63 |
| Lou et al. 2005[167] | 1 week post-intervention | Chemotherapy | Anxiety | 45.4 | 8.8 | 34.7 | 8.3 |
| Liu et al. 2006[143] | Post-intervention | Radiofrequency heat therapy | Depression | 49.01 | 8.56 | 37.68 | 4.89 |
|  |  |  | Anxiety | 56.12 | 7.43 | 35.36 | 5.89 |
| Cheng et al.2006[107] | Post-chemotherapy | Chemotherapy | Depression | 20.33 | 4.86 | 10.80 | 3.55 |
|  |  |  | Anxiety | 19.80 | 4.71 | 10.53 | 2.99 |
| Wang et al.2006[75] | 8 weeks post-intervention | - | Depression | 45.46 | 10.45 | 42.14 | 10.02 |
|  |  |  | Anxiety | 46.41 | 7.44 | 36.45 | 9.23 |
| Ni et al. 2007[168] | 1 week post-chemotherapy | Chemotherapy | Anxiety | 58.87 | 3.26 | 57.13 | 3.21 |
| Pang&Wang 2007[169] | 1 day pre-surgery | Surgery | Anxiety | 47 | 7 | 41 | 7 |
| Qian&Cai 2007[50] | Post- chemotherapy | Chemotherapy | Depression | 55.76 | 6.67 | 45.55 | 6.88 |
|  |  |  | Anxiety | 51.58 | 6.69 | 40.29 | 5.89 |
| Wen&Liang 2007[69] | 1 week post-chemotherapy | Chemotherapy | Depression | 46.39 | 10.26 | 41.23 | 9.91 |
|  |  |  | Anxiety | 46.96 | 9.99 | 37.27 | 9.88 |
| Kang 2007[128] | Post-intervention | - | Depression | 68.50 | 3.44 | 66.30 | 5.31 |
|  |  |  | Anxiety | 55.87 | 2.54 | 53.70 | 4.74 |
| Zheng et al. 2007[109] | 2 weeks after the first assessment | - | Depression | 48.89 | 9.14 | 44.13 | 8.41 |
|  |  |  | Anxiety | 46.94 | 8.92 | 43.41 | 9.23 |
| Deng et al. 2007[110] | 1 week post-intervention | Chemotherapy | Depression | 54.05 | 5.57 | 43.87 | 4.28 |
|  |  |  | Anxiety | 58.33 | 4.76 | 46.97 | 6.43 |
| Xing 2007[103] | 3 days post-surgery | Surgery | Depression | 45.48 | 3.98 | 41.36 | 5.02 |
|  |  |  | Anxiety | 46.23 | 7.25 | 40.27 | 6.64 |
| Wu et al. 2007[59] | 2 weeks after the third chemo | Chemotherapy | Depression | 58.88 | 5.28 | 55.18 | 5.35 |
|  |  |  | Anxiety | 56.53 | 4.61 | 53.28 | 4.23 |
| Xu 2007[88] | Post-surgery | Surgery | Depression | 43.8 | 2.6 | 39.8 | 3.2 |
|  |  |  | Anxiety | 42.6 | 2.6 | 38.8 | 2.9 |
| Han&Liu 2007[151] | 4 weeks after hospitalization | - | Depression | 47.97 | 10.75 | 40.35 | 9.47 |
|  |  |  | Anxiety | 46.75 | 9.42 | 40.64 | 9.87 |
| Huang et al.2008[54] | 1 day pre-surgery | - | Depression | 48.45 | 9.47 | 39.68 | 9.02 |
|  |  |  | Anxiety | 48.98 | 5.96 | 40.38 | 5.37 |
| Zheng et al. 2008[116] | Post-radiotherapy | Radiotherapy | Depression | 50.59 | 12.43 | 43.76 | 9.22 |
|  |  |  | Anxiety | 47.05 | 10.38 | 41.16 | 8.62 |
| Yang 2008[79] | 12 weeks post-intervention | Chemotherapy | Depression | 41.16 | 6.71 | 36.26 | 7.19 |
|  |  |  | Anxiety | 42.11 | 5.56 | 34.34 | 6.43 |
| Han 2008[163] | 1 day pre-surgery | Surgery | Anxiety | 45.72 | 2.53 | 41.24 | 3.27 |
| Jiang et al. 2008[164] | Post-intervention | Chemotherapy | Anxiety | 62.21 | 9.20 | 45.42 | 6.47 |
| Li et al.2008[166] | 30 min pre-surgery | Surgery | Anxiety | 37.55 | 4.59 | 27.2 | 3.54 |
| Wang et al.2008[172] | Post-intervention | Surgery | Anxiety | 44.33 | 9.41 | 34.80 | 8.53 |
| Ji 2008[106] | 4 weeks after hospitalization | - | Depression | 48.19 | 10.79 | 40.57 | 9.51 |
| Jin & Zhu 2008[122] | Post-chemotherapy | Chemotherapy | Depression | 51.4 | 4.4 | 42.1 | 2.5 |
|  |  |  | Anxiety | 52.8 | 4.8 | 44.1 | 3.8 |
| Li et al.2008[99] | 4 weeks post-intervention | Chemotherapy | Depression | 35.12 | 4.45 | 31.57 | 4.48 |
|  |  |  | Anxiety | 38.17 | 5.93 | 33.90 | 4.78 |
| Liu et al. 2008[52] | 6 months post-surgery | Surgery | Depression | 59.5 | 6.78 | 48.8 | 5.43 |
|  |  |  | Anxiety | 48.3 | 4.88 | 41.3 | 6.34 |
| Yang 2008[136] | 8 weeks post-treatment | Chemotherapy | Depression | 50.15 | 5.82 | 45.08 | 7.25 |
|  |  |  | Anxiety | 49.00 | 7.07 | 42.23 | 4.72 |
| Zhou 2008[100] | Post-intervention | Chemotherapy | Depression | 55.6 | 9.6 | 47.7 | 7.1 |
|  |  |  | Anxiety | 57.5 | 7.5 | 53.0 | 5.8 |
| Mao et al.2008[113] | Hospital discharge | - | Depression | 19.47 | 9.57 | 15.62 | 8.12 |
|  |  |  | Anxiety | 14.35 | 4.39 | 9.78 | 7.33 |
| Liu2008[132] | Before the third chemotherapy | Surgery/  Chemotherapy | Depression | 10.22 | 3.52 | 5.06 | 2.60 |
|  |  |  | Anxiety | 10.83 | 5.61 | 4.35 | 3.05 |
| Zheng et al. 2008[125] | 2 weeks post-chemotherapy | Chemotherapy | Depression | 59.68 | 6.27 | 54.68 | 3.57 |
|  |  |  | Anxiety | 56.34 | 5.38 | 53.06 | 4.16 |
| Chen et al.2009[159] | 14 days post-radiotherapy | Surgery | Anxiety | 50.21 | 5.11 | 45.11 | 6.39 |
| Li 2009[89] | Post-chemotherapy | Chemotherapy | Depression | 57 | 8 | 47 | 7 |
|  |  |  | Anxiety | 52 | 8 | 41 | 7 |
| Li et al.2009[78] | 5 days Post-surgery | Surgery | Depression | 44.0 | 4.7 | 36.4 | 4.0 |
|  |  |  | Anxiety | 40.6 | 3.8 | 32.2 | 3.1 |
| Fu et al. 2009[145] | After the fourth chemotherapy | Chemotherapy | Depression | 38.74 | 7.39 | 35.64 | 7.80 |
|  |  |  | Anxiety | 26.18 | 5.48 | 24.90 | 5.52 |
| Qiu 2009[133] | Before the second chemotherapy | Chemotherapy | Depression | 54.87 | 4.369 | 43.57 | 5.257 |
|  |  |  | Anxiety | 49.60 | 4.782 | 40.90 | 4.559 |
| Sun 2009[134] | 4 weeks post-intervention | Radiotherapy | Depression | 8.32 | 2.471 | 7.87 | 2.862 |
|  |  |  | Anxiety | 7.93 | 3.732 | 7.23 | 4.135 |
| Xia 2009[118] | 7 days after hospitalization | - | Depression | 51.26 | 4.88 | 47.98 | 4.96 |
|  |  |  | Anxiety | 49.12 | 5.48 | 44.56 | 5.36 |
| Zhang 2009[139] | 6 weeks post-intervention | - | Depression | 53.18 | 4.10 | 48.79 | 5.43 |
| Zhou 2009[140] | Post-intervention | Chemotherapy | Depression | 42.87 | 6.34 | 35.60 | 5.62 |
|  |  |  | Anxiety | 44.50 | 7.69 | 37.90 | 5.51 |
| Li et al. 2009[63] | 1 month post-discharge | Radiotherapy | Depression | 43.29 | 4.98 | 38.90 | 4.35 |
|  |  |  | Anxiety | 42.06 | 5.22 | 37.31 | 5.09 |
| Geng et al 2010[104] | 4 weeks after hospitalization | - | Depression | 54.0 | 9.32 | 41.5 | 6.58 |
|  |  |  | Anxiety | 59.6 | 7.48 | 52.3 | 8.80 |
| Zhan&Cheng2010[105] | Post-intervention | Chemotherapy | Depression | 51.52 | 10.96 | 50.25 | 7.32 |
|  |  |  | Anxiety | 51.06 | 10.16 | 46.28 | 8.56 |
| Cheng et al.2010[45] | 1 month post-intervention | - | Depression | 48.52 | 4.17 | 42.04 | 4.68 |
|  |  |  | Anxiety | 46.46 | 5.03 | 35.52 | 3.45 |
| Li et al. 2010[91] | 4 weeks post-intervention | Surgery | Depression | 51.62 | 9.51 | 42.33 | 7.12 |
|  |  |  | Anxiety | 56.29 | 7.28 | 36.85 | 6.81 |
| Guan et al. 2010[81] | 6 months post-treatment | Chemotherapy | Depression | 39.8 | 7.4 | 25.2 | 4.2 |
|  |  |  | Anxiety | 36.4 | 7.8 | 25.5 | 3.4 |
| Li 2010[111] | 3 months post-chemotherapy | Chemotherapy | Depression | 53.9 | 12.1 | 39.6 | 9.3 |
|  |  |  | Anxiety | 52.6 | 10.7 | 41.4 | 10.8 |
| Zhang 2010[138] | 8 weeks post-intervention | - | Depression | 39.59 | 6.82 | 35.51 | 5.14 |
|  |  |  | Anxiety | 36.88 | 4.51 | 32.92 | 4.03 |
| Su & Wang2010[170] | 30-60 min pre-surgery | Surgery | Anxiety | 51 | 8.441 | 42.048 | 8.405 |
| Fu et al. 2010[162] | Post-intervention | - | Anxiety | 45.14 | 5.49 | 42.81 | 5.36 |
| Wu & Zhang2010[173] | 3 days post-intervention | - | Anxiety | 55.67 | 8.85 | 42.31 | 8.66 |
| Zhou 2010[179] | The evening before surgery | Surgery | Anxiety | 66 | 3.78 | 55 | 4.10 |
| You et al. 2010[174] | 2 weeks post-intervention | Chemotherapy | Anxiety | 33.45 | 2.33 | 30.17 | 2.12 |
| Ren et al. 2010[154] | 1 month post-intervention | - | Depression | 53.84 | 12.76 | 40.40 | 7.89 |
|  |  |  | Anxiety | 48.46 | 9.69 | 35.93 | 6.59 |
| Xu 2010[40] | Hospital discharge | Surgery | Depression | 54.38 | 4.63 | 44.17 | 4.32 |
|  |  |  | Anxiety | 57.12 | 5.17 | 45.21 | 5.21 |
| Guo et al.2010[71] | 3 months post-treatment | - | Depression | 55.56 | 4.13 | 53.45 | 3.59 |
|  |  |  | Anxiety | 58.36 | 4.23 | 54.45 | 5.18 |
| Tang et al. 2010[126] | 3 months post-chemotherapy | Chemotherapy | Depression | 41.457 | 8.304 | 28.387 | 4.256 |
|  |  |  | Anxiety | 40.872 | 7.964 | 34.577 | 4.851 |
| Liu et al. 2010[46] | Post-intervention | Surgery | Depression | 40.75 | 8.49 | 35.60 | 8.00 |
|  |  |  | Anxiety | 42.96 | 10.11 | 34.12 | 9.86 |
| Shi et al.2010[108] | 6 weeks post-treatment | - | Depression | 11.32 | 1.23 | 8.23 | 1.12 |
| Liu et al. 2010[87] | 8 weeks post-chemotherapy | Chemotherapy | Depression | 38 | 8 | 25 | 9 |
|  |  |  | Anxiety | 49 | 6 | 26 | 6 |
| Wang 2010[90] | Post-intervention | Chemotherapy | Depression | 42.58 | 9.39 | 29.74 | 6.42 |
|  |  |  | Anxiety | 36.17 | 5.86 | 28.49 | 4.72 |
| Huang et al. 2010[86] | 3 months post-discharge | Chemotherapy | Depression | 32.25 | 8.12 | 30.12 | 5.12 |
|  |  |  | Anxiety | 34.52 | 5.79 | 33.87 | 4.56 |
| Zhang & Yu 2011[94] | Post-chemotherapy | Chemotherapy | Depression | 47.22 | 4.64 | 40.54 | 3.69 |
|  |  |  | Anxiety | 41 | 3.03 | 37.46 | 4.25 |
| Du et al. 2011[61] | 6 months post-intervention | Chemotherapy | Depression | 45.87 | 9.25 | 41.43 | 5.62 |
|  |  |  | Anxiety | 45.15 | 4.68 | 42.21 | 4.83 |
| Li et al. 2011[149] | 1 day pre-discharge | Surgery | Depression | 53.75 | 2.00 | 47.84 | 3.03 |
|  |  |  | Anxiety | 51.55 | 1.85 | 43.55 | 3.41 |
| Zhou et al. 2011[156] | The third time of hospitalization | Surgery | Depression | 32.15 | 3.86 | 25.67 | 2.74 |
| Liu 2011[131] | 14 days post-surgery | Surgery | Depression | 49.97 | 14.93 | 38.80 | 12.95 |
|  |  |  | Anxiety | 44.87 | 8.23 | 38.70 | 10.95 |
| Shen et al.2011[64] | Post-surgery | Surgery | Depression | 45.4 | 10.9 | 40.3 | 9.5 |
|  |  |  | Anxiety | 48.0 | 10.6 | 46.3 | 10.2 |
| Zhu et al.2011[65] | 2 months post-intervention | - | Depression | 46.45 | 6.94 | 38.42 | 8.03 |
| Meng et al.2011[95] | Post-intervention | - | Depression | 51.87 | 9.01 | 42.98 | 9.05 |
|  |  |  | Anxiety | 49.32 | 9.01 | 42.96 | 9.08 |
| Dai et al.2011[160] | 1 day pre-discharge | Radiotherapy | Anxiety | 44.72 | 9.82 | 31.00 | 5.50 |
| Jiao et al. 2011[165] | 30-60 min pre-surgery | Surgery | Anxiety | 52.94 | 4.16 | 42.44 | 4.49 |
| Ye 2011[56] | Post-intervention | Surgery | Depression | 50.8 | 10.5 | 43.2 | 8.4 |
|  |  |  | Anxiety | 50.7 | 9.1 | 41.6 | 6.65 |
| Li 2011[130] | 9 days post-surgery | Surgery | Depression | 59.7 | 7.5 | 52.3 | 5.8 |
| Liu et al. 2011[41] | - | Chemotherapy | Depression | 43.42 | 7.48 | 39.84 | 5.18 |
| Wang et al. 2011[37] | 8 weeks post-radiotherapy | Radiotherapy | Depression | 16.09 | 8.62 | 11.53 | 4.52 |
|  |  |  | Anxiety | 12.32 | 7.24 | 8.67 | 3.72 |
| Cao2011[176] | 6 days post-surgery | Surgery | Anxiety | 38.19 | 8.11 | 30.13 | 6.69 |
| Zhao&Zhang2011[177] | Post-intervention | Surgery | Anxiety | 52.81 | 6.39 | 43.97 | 6.36 |
| Cao & Li 2011[67] | 8 weeks post-intervention | - | Depression | 45.90 | 10.96 | 42.01 | 8.01 |
|  |  |  | Anxiety | 36.99 | 6.2 | 34.01 | 6.0 |
| Huang et al. 2011[152] | 4 weeks post-intervention | - | Depression | 56.00 | 12.00 | 51.00 | 3.00 |
| Hu & Yan2011[62] | Post-intervention | - | Depression | 41.18 | 4.74 | 37.15 | 7.51 |
|  |  |  | Anxiety | 39.41 | 11.53 | 33.82 | 4.93 |
| Guan & Jie 2011[120] | 2 weeks post-chemotherapy | Chemotherapy | Depression | 58.89 | 7.87 | 43.24 | 7.42 |
|  |  |  | Anxiety | 55.98 | 8.19 | 46.14 | 6.23 |
| Lv et al. 2011[77] | 1 month post-surgery | Surgery | Depression | 50.0 | 3.87 | 36.7 | 5.13 |
|  |  |  | Anxiety | 41.17 | 5.07 | 36.73 | 4.72 |
| Li et al. 2011[182] | The third time of hospitalization | Surgery | Anxiety | 40.35 | 4.44 | 30.87 | 2.71 |
| Cao&Jiang2011[180] | 4 weeks post-intervention | - | Anxiety | 48.22 | 6.15 | 38.36 | 6.08 |
| Huang2011[102] | 8 weeks post-treatment | Chemotherapy | Depression | 25 | 4.78 | 13.20 | 4.31 |
| Zheng et al. 2011[141] | 21 days post-intervention | Chemotherapy | Depression | 42.91 | 10.09 | 35.93 | 7.87 |
|  |  |  | Anxiety | 40.03 | 8.67 | 33.12 | 6.33 |
| Wu & Dong2011[47] | 8 weeks post-treatment | - | Depression | 20.19 | 7.13 | 16.32 | 6.87 |
|  |  |  | Anxiety | 13.23 | 5.66 | 9.89 | 4.33 |
| Zheng et al. 2012[150] | 1 day before the end of chemo | Chemotherapy | Depression | 52.82 | 6.38 | 43.98 | 6.37 |
| Wang&Xiao 2012[124] | 8 weeks post-treatment | - | Depression | 50 | 7 | 35 | 6 |
|  |  |  | Anxiety | 57 | 6 | 39 | 7 |
| Wei 2012[135] | 1 week post-intervention | Endocrinotherapy | Depression | 56.97 | 5.66 | 51.50 | 4.07 |
|  |  |  | Anxiety | 55.63 | 6.07 | 50.37 | 6.26 |
| Feng 2012[55] | post-treatment | - | Depression | 53.6 | 7.9 | 45.3 | 8.7 |
|  |  |  | Anxiety | 53.6 | 8.7 | 45.1 | 9.6 |
| Yang et al. 2012[73] | Post-surgery | Surgery | Depression | 37.60 | 2.82 | 33.05 | 2.46 |
|  |  |  | Anxiety | 39.75 | 3.67 | 35.95 | 3.07 |
| Zhao et al. 2012[84] | 6 weeks post-treatment | - | Depression | 57.67 | 9.78 | 45.0 | 10.05 |
|  |  |  | Anxiety | 57.62 | 10.02 | 44.98 | 9.72 |
| Gu 2012[121] | Post-intervention | Chemotherapy | Depression | 45.7 | 6.8 | 40.6 | 5.9 |
|  |  |  | Anxiety | 43.1 | 4.5 | 36.7 | 5.9 |
| Zheng2012[58] | 6 weeks post-intervention | - | Depression | 30.33 | 8.17 | 19.81 | 6.51 |
|  |  |  | Anxiety | 19.52 | 5.22 | 15.55 | 5.14 |
| Yang2012[66] | Post-intervention | - | Depression | 44.53 | 6.74 | 31.88 | 9.37 |
| Sun et al. 2012[60] | Post- chemotherapy | Chemotherapy | Depression | 59.45 | 4.11 | 52.69 | 3.46 |
|  |  |  | Anxiety | 55.42 | 5.18 | 48.15 | 4.63 |
| Liu et al.2012[98] | Post-intervention | - | Depression | 49.32 | 8.61 | 42.01 | 8.84 |
|  |  |  | Anxiety | 51.23 | 9.54 | 45.07 | 9.87 |
| Yang et al. 2012[70] | Post-treatment | - | Depression | 52.4 | 5.0 | 40.7 | 4.1 |
|  |  |  | Anxiety | 53.3 | 5.1 | 41.2 | 4.8 |
| Li 2012[129] | Hospital discharge | Microwave ablation | Depression | 15.58 | 4.213 | 11.85 | 4.324 |
|  |  |  | Anxiety | 53.14 | 7.524 | 47.39 | 7.576 |
| Zhu & Hu 2012[68] | Post-intervention | Surgery | Depression | 50.08 | 7.91 | 45.18 | 6.25 |
|  |  |  | Anxiety | 52.32 | 6.99 | 41.66 | 6.68 |
| Liu 2012[48] | Post-treatment | - | Depression | 20.11 | 3.6 | 16.88 | 4.25 |
| Shi et al. 2012[92] | 2 weeks after hospitalization | Surgery | Depression | 53.22 | 4.36 | 49.89 | 4.83 |
|  |  |  | Anxiety | 52.58 | 6.14 | 47.73 | 5.24 |
| Jia2012[127] | Post-surgery | Surgery | Depression | 9.68 | 2.72 | 5.16 | 1.66 |
|  |  |  | Anxiety | 15.78 | 4.41 | 9.61 | 2.70 |
| Zhang 2012[97] | 1 month post-discharge | - | Depression | 63.67 | 8.18 | 52.49 | 7.30 |
|  |  |  | Anxiety | 64.52 | 7.85 | 53.76 | 5.17 |
| Chen 2012[51] | Hospital discharge | Chemotherapy | Depression | 55.51 | 7.41 | 46.54 | 6.92 |
|  |  |  | Anxiety | 52.14 | 7.40 | 41.22 | 6.81 |
| Li et al.2012[146] | 50 days post-radiotherapy | Radiotherapy | Depression | 15.30 | 3.52 | 12.50 | 1.56 |
|  |  |  | Anxiety | 10.80 | 3.09 | 8.28 | 2.05 |
| Yang&Wang2012[83] | 7 days post-chemotherapy | Chemotherapy | Depression | 19.33 | 3.65 | 9.80 | 3.32 |
|  |  |  | Anxiety | 19.80 | 4.71 | 9.53 | 2.99 |
| Jiang et al.2012[49] | 12 months post-treatment | Surgery | Depression | 48.8 | 7.6 | 38.9 | 8.8 |
| Fan&Pan 2012[161] | 3 months post-discharge | Radiotherapy/  Chemotherapy | Anxiety | 60.368 | 9.581 | 45.211 | 11.193 |
| Li et al. 2012[72] | Post-surgery | Surgery | Depression | 50.26 | 7.45 | 41.28 | 6.48 |
|  |  |  | Anxiety | 43.74 | 10.23 | 38.23 | 7.28 |
| Han et al. 2012[85] | 2 weeks post-treatment | - | Depression | 67.68 | 8.23 | 55.05 | 6.75 |
|  |  |  | Anxiety | 67.62 | 9.08 | 51.95 | 7.56 |
| Zheng et al. 2012[178] | Post-intervention | Surgery | Anxiety | 51.45 | 8.23 | 40.87 | 5.98 |
| Yuan&Wu 2013[147] | 2 weeks post-treatment | - | Depression | 66.32 | 5.18 | 59.24 | 5.32 |
|  |  |  | Anxiety | 70.25 | 4.51 | 61.35 | 5.24 |
| Zhu et al. 2013[36] | Hospital discharge | Chemotherapy | Depression | 45.89 | 7.12 | 31.36 | 6.36 |
|  |  |  | Anxiety | 47.76 | 7.32 | 30.78 | 6.56 |
| Du 2013[74] | Post-intervention | - | Depression | 41.29 | 5.08 | 31.62 | 5.38 |
|  |  |  | Anxiety | 43.12 | 2.65 | 38.12 | 2.85 |
| Mu et al. 2012[181] | 1 day pre-surgery | Surgery | Anxiety | 38.49 | 3.78 | 34.68 | 3.17 |
| Liu & Gan 2013[43] | Post-examination | - | Depression | 49.89 | 9.57 | 41.68 | 4.88 |
|  |  |  | Anxiety | 54.41 | 6.43 | 39.12 | 5.83 |
| Zhang 2013[96] | 1 day pre-discharge | - | Depression | 47.75 | 5.614 | 43.04 | 5.63 |
|  |  |  | Anxiety | 46.74 | 5.99 | 40.93 | 5.35 |
| Zhang et al. 2013[142] | 28 days post-intervention | Chemotherapy | Depression | 42.80 | 10.11 | 35.86 | 7.61 |
|  |  |  | Anxiety | 40.10 | 8.57 | 33.13 | 6.34 |
| Guo et al.2013[155] | 2 weeks post-radiotherapy | Radiotherapy | Depression | 59.05 | 9.41 | 51.48 | 7.54 |
|  |  |  | Anxiety | 55.69 | 10.01 | 48.78 | 8.95 |
| Liu 2013[119] | 1 month post-intervention | - | Depression | 31.6 | 8.1 | 20.7 | 5.1 |
|  |  |  | Anxiety | 41.8 | 7.8 | 29.7 | 6.1 |
| Zhai et al. 2013[82] | 6 weeks post-intervention | - | Depression | 55.38 | 8.96 | 42.29 | 8.92 |
|  |  |  | Anxiety | 60.46 | 8.25 | 44.26 | 8.11 |
| Ci et al. 2013[117] | Post-intervention | Chemotherapy | Depression | 48.93 | 4.78 | 45.14 | 4.57 |
|  |  |  | Anxiety | 48.12 | 5.60 | 44.36 | 5.48 |
| Liu 2013[112] | Post-intervention | - | Depression | 45.79 | 7.13 | 37.15 | 7.24 |
|  |  |  | Anxiety | 46.13 | 6.15 | 37.55 | 7.29 |
| Liu et al.2013[57] | Hospital discharge | Radiotherapyaaw | Depression | 42.3 | 3.1 | 37.6 | 3.2 |
|  |  |  | Anxiety | 41.8 | 3.2 | 36.9 | 2.4 |
| Qiu et al.2013[157] | 6 months post-intervention | - | Depression | 14.35 | 5.21 | 7.51 | 3.71 |
|  |  |  | Anxiety | 43.10 | 10.09 | 37.74 | 5.20 |
| Mao et al. 2013[80] | 8 weeks post-intervention | - | Depression | 58.5 | 9.3 | 47.9 | 8.1 |
|  |  |  | Anxiety | 53.1 | 9.5 | 41.9 | 8.0 |
| Zhang 2013[114] | 2 weeks post-surgery | Surgery | Depression | 58.7 | 9.2 | 49.5 | 8.7 |
|  |  |  | Anxiety | 54.3 | 9.9 | 48.2 | 9.2 |
| Yu 2013[137] | 1 year post-discharge | Radiotherapy | Depression | 40.14 | 5.99 | 32.12 | 3.18 |
|  |  |  | Anxiety | 39.58 | 4.18 | 36.33 | 6.18 |
| Wang 2013[53] | Post-intervention | - | Depression | 43.5 | 10.6 | 39.6 | 8.9 |
|  |  |  | Anxiety | 41.2 | 11.2 | 36.6 | 8.9 |
| Tian et al. 2013[171] | 5 days after hospitalization | - | Anxiety | 75.47 | 12.04 | 61.14 | 14.31 |
| Yu 2013[175] | The morning of surgery | Surgery | Anxiety | 56.91 | 8.72 | 42.37 | 3.76 |

Abbreviations: SD, standard deviation; Mean1/SD1, the mean scores/ standard deviation of depression/anxiety in control group; Mean2/SD2, the mean scores/ standard deviation of depression/anxiety in experimental group; -, no report.
