# Supplementary figures and images for: The effects of psychological interventions on depression and anxiety among Chinese adults with cancer: a meta-analysis of randomized controlled studies
Source: BMC Cancer. 2014 Dec 15;14:956. doi: 10.1186/1471-2407-14-956 (PMC4301929; doi:10.1186/1471-2407-14-956)

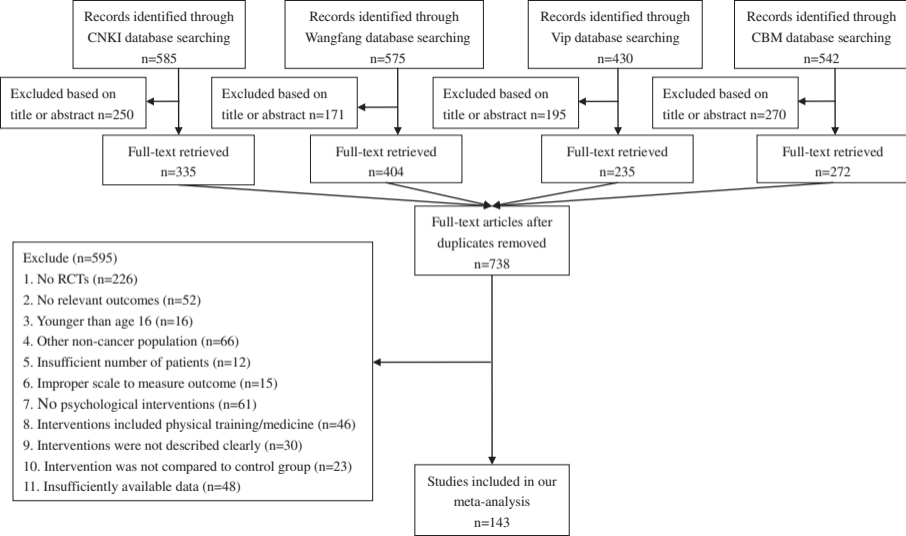

Supplement: Supplementary file 3 — Authors’ original file for figure 1 [file 12885_2014_5105_MOESM3_ESM.pdf]

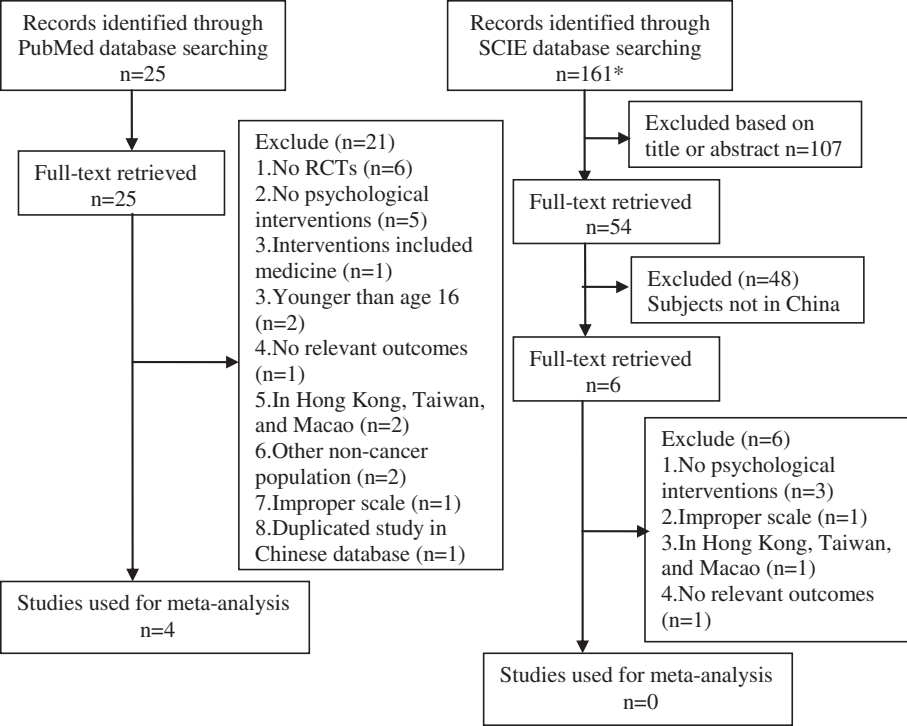

Supplement: Supplementary file 4 — Authors’ original file for figure 2 [file 12885_2014_5105_MOESM4_ESM.pdf]

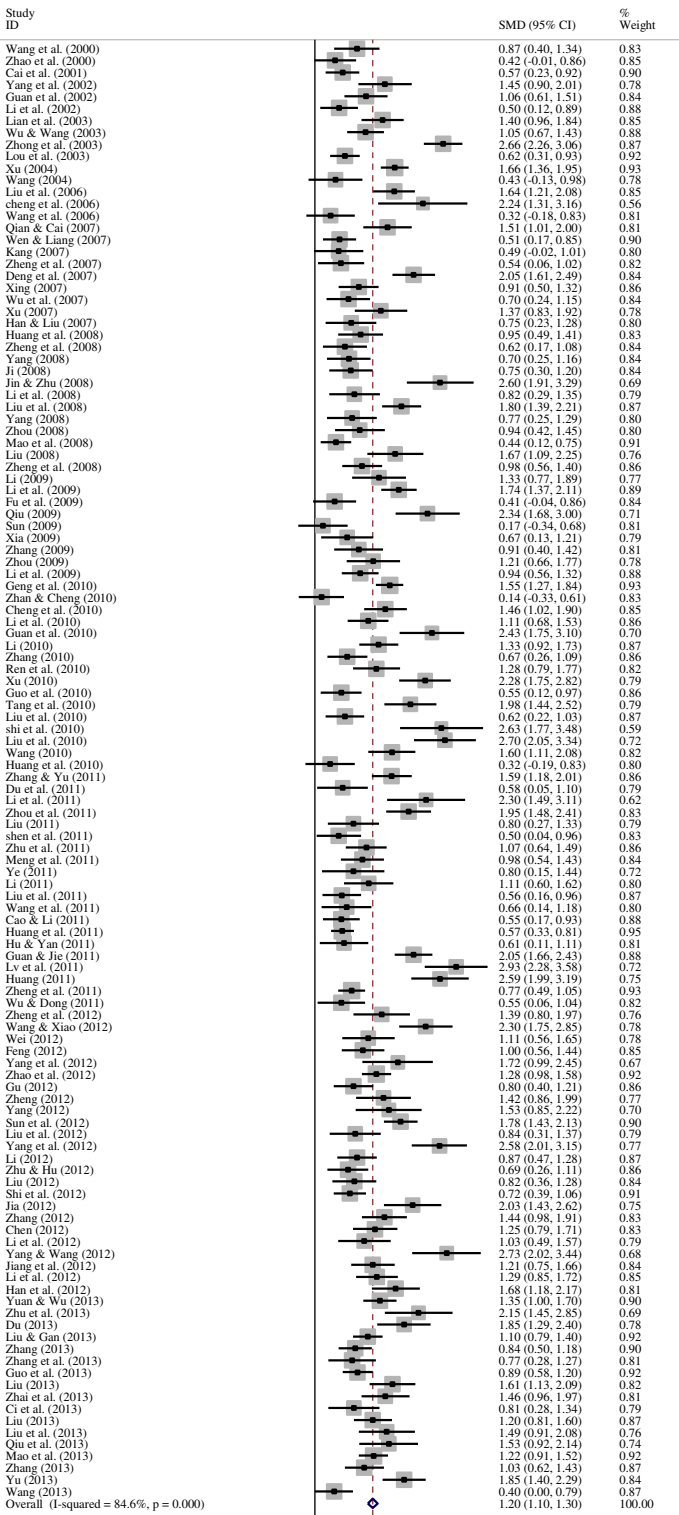

0 .5 .81 Cohen's d

Favors control group Favors experimental group

Supplement: Supplementary file 5 — Authors’ original file for figure 3 [file 12885_2014_5105_MOESM5_ESM.pdf]

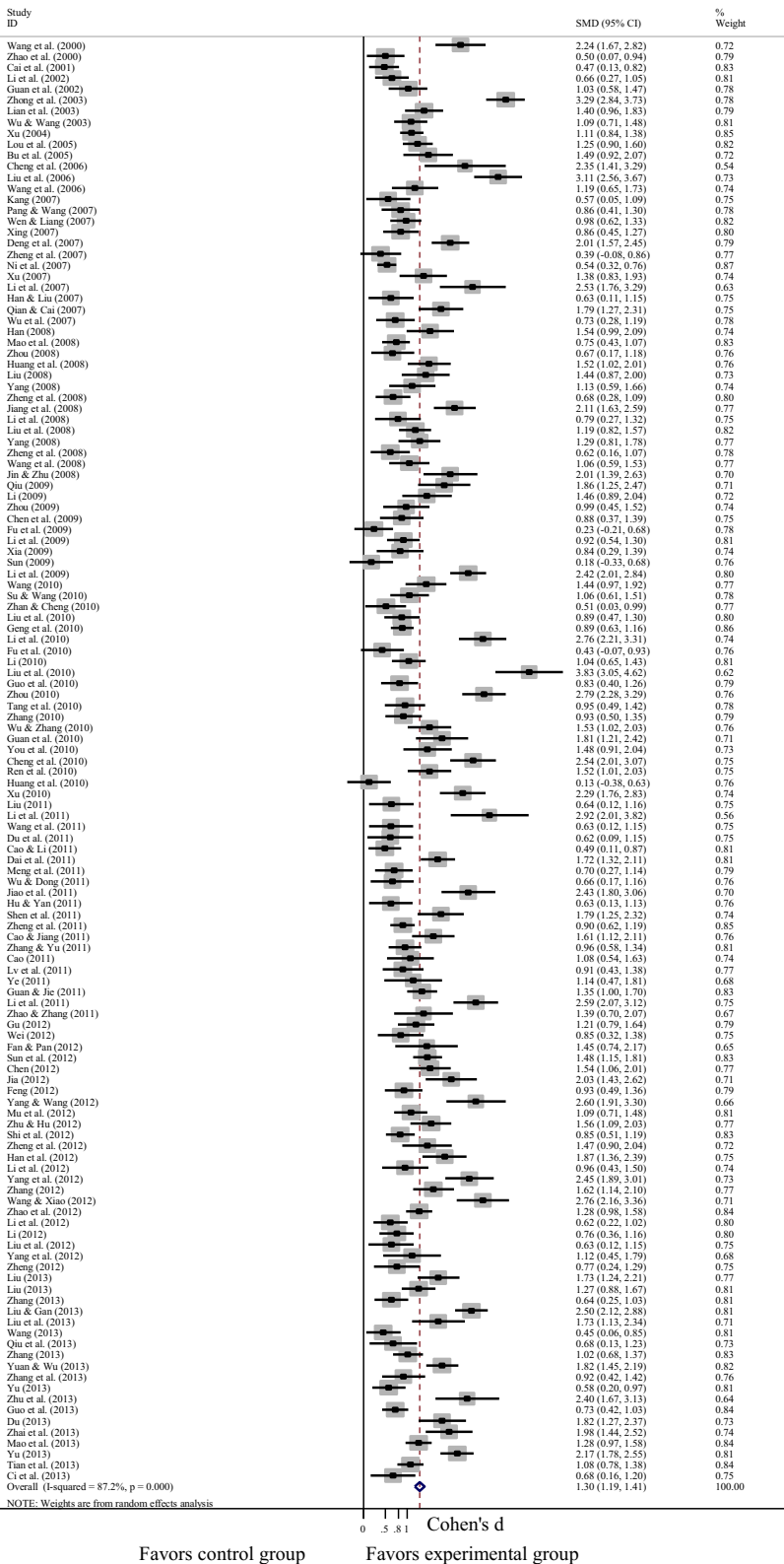

Supplement: Supplementary file 6 — Authors’ original file for figure 4 [file 12885_2014_5105_MOESM6_ESM.pdf]

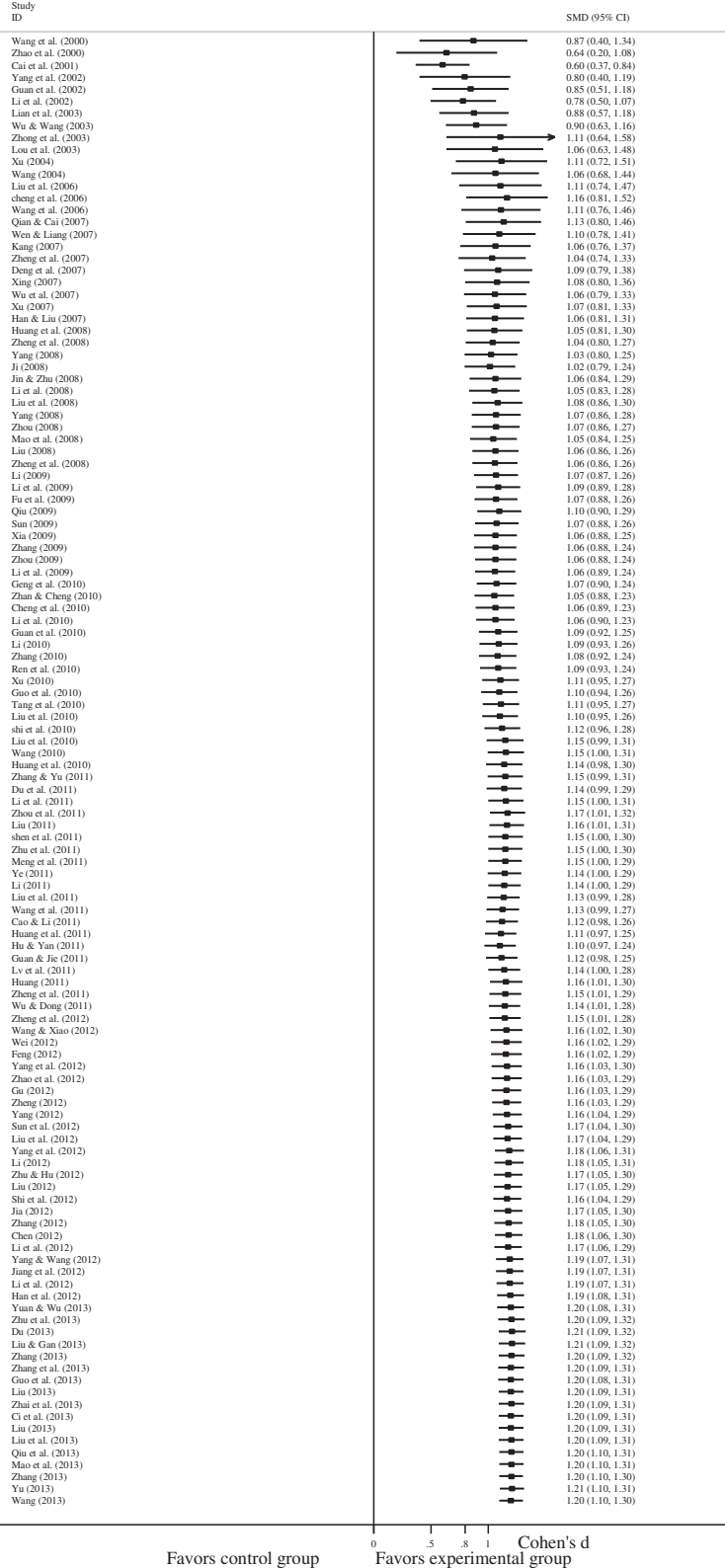

Supplement: Supplementary file 7 — Authors’ original file for figure 5 [file 12885_2014_5105_MOESM7_ESM.pdf]

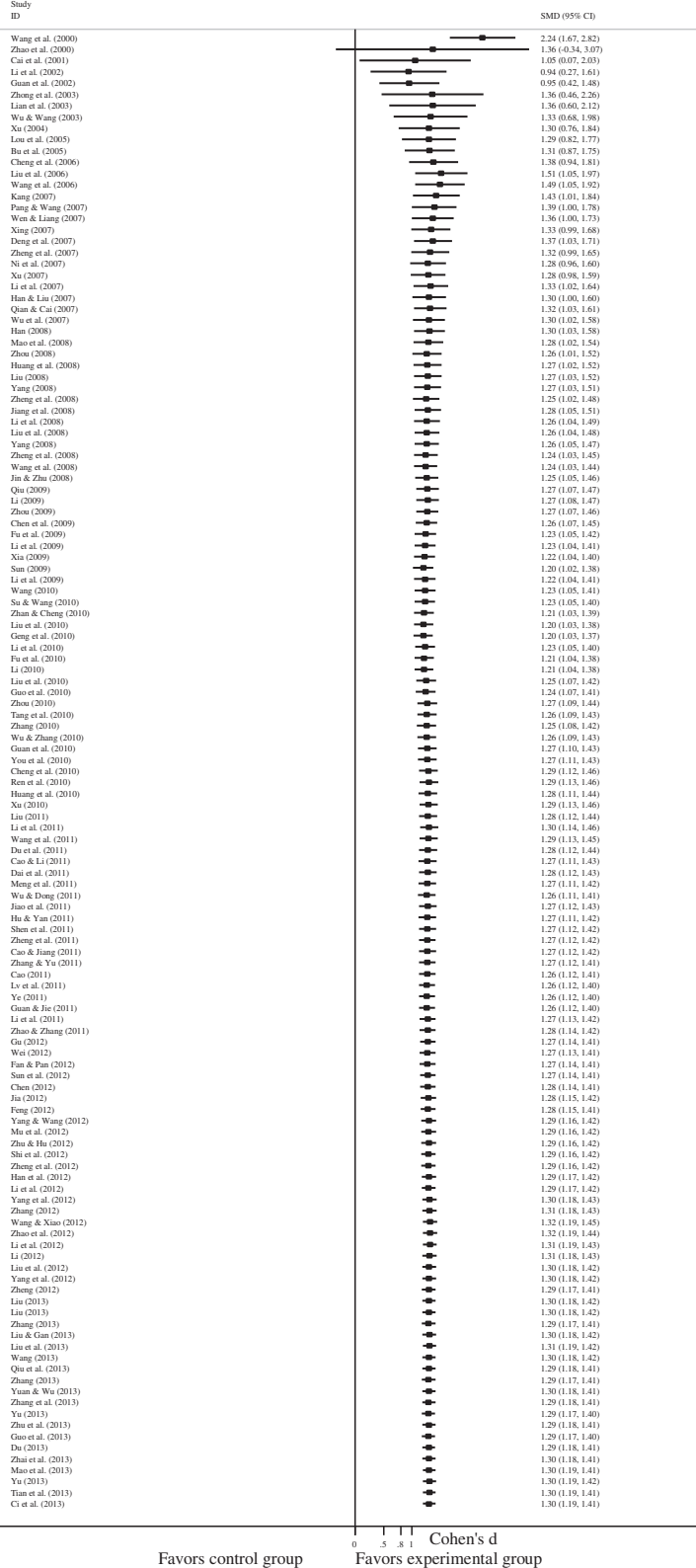

Supplement: Supplementary file 8 — Authors’ original file for figure 6 [file 12885_2014_5105_MOESM8_ESM.pdf]
